# Long non-coding RNA GBCDRlnc1 induces chemoresistance of gallbladder cancer cells by activating autophagy

**DOI:** 10.1186/s12943-019-1016-0

**Published:** 2019-04-05

**Authors:** Qiang Cai, Shouhua Wang, Longyang Jin, Mingzhe Weng, Di Zhou, Jiandong Wang, Zhaohui Tang, Zhiwei Quan

**Affiliations:** 1 Department of General Surgery, XinHua Hospital, Shanghai JiaoTong University School of Medicine, Shanghai, 200092 China; 2 Department of Surgery, Shanghai Institute of Digestive Surgery, Ruijin Hospital, Shanghai JiaoTong University School of Medicine, Shanghai, 200025 China

**Keywords:** lncRNA GBCDRlnc1, Gallbladder cancer, Chemoresistance, Autophagy, PGK1

## Abstract

**Background:**

Gallbladder cancer is the most common biliary tract malignancy and not sensitive to chemotherapy. Autophagy is an important factor prolonging the survival of cancer cells under chemotherapeutic stress. We aimed to investigate the role of long non-coding RNAs (lncRNAs) in autophagy and chemoresistance of gallbladder cancer cells.

**Methods:**

We established doxorubicin (Dox)-resistant gallbladder cancer cells and used microarray analysis to compare the expression profiles of lncRNAs in Dox-resistant gallbladder cancer cells and their parental cells. Knockdown or exogenous expression of lncRNA combined with in vitro and in vivo assays were performed to prove the functional significance of lncRNA. The effects of lncRNA on autophagy were assessed by stubRFP-sensGFP-LC3 and western blot. We used RNA pull-down and mass spectrometry analysis to identify the target proteins of lncRNA.

**Results:**

The drug-resistant property of gallbladder cancer cells is related to their enhanced autophagic activity. And we found a lncRNA ENST00000425894 termed gallbladder cancer drug resistance-associated lncRNA1 (GBCDRlnc1) that serves as a critical regulator in gallbladder cancer chemoresistance. Furthermore, we discovered that GBCDRlnc1 is upregulated in gallbladder cancer tissues. Knockdown of GBCDRlnc1, via inhibiting autophagy at initial stage, enhanced the sensitivity of Dox-resistant gallbladder cancer cells to Dox in vitro and in vivo. Mechanically, we identified that GBCDRlnc1 interacts with phosphoglycerate kinase 1 and inhibits its ubiquitination in Dox-resistant gallbladder cancer cells, which leads to the down-regulation of autophagy initiator ATG5-ATG12 conjugate.

**Conclusions:**

Our findings established that the chemoresistant driver GBCDRlnc1 might be a candidate therapeutic target for the treatment of advanced gallbladder cancer.

**Electronic supplementary material:**

The online version of this article (10.1186/s12943-019-1016-0) contains supplementary material, which is available to authorized users.

## Introduction

Gallbladder cancer is the most common biliary tract malignancy and the fifth most common digestive tract malignancy worldwide [1]. Owing to the vague and nonspecific symptoms and signs, the majority of gallbladder cancer patients are diagnosed at an advanced stage with extremely poor prognosis [2]. In this setting, surgical curative resection provides quite marginal benefits. The adjuvant therapy that based on chemotherapy is considered a chance to prolong the survival [3]. However, previous studies corroborated that gallbladder cancer is not sensitive to chemotherapeutic drugs [4, 5]. To this end, the present study was aimed to explore the molecular mechanisms associated with gallbladder cancer chemoresistance and figure out the way to improve the outcomes.

Long non-coding RNAs (lncRNAs), a class of transcripts longer than 200 bases without protein-coding potential, are regarded as transcriptional ‘noise’ initially. In recent years, lncRNAs have been frequently reported to play orchestrated roles in multiple biological processes of cancer progression, including proliferation, apoptosis, epithelial-mesenchymal transition, or autophagy [6–8]. Although autophagy is a double-edged sword in tumorigenesis, accumulating evidence supported that cytoprotective autophagy that confers stress tolerance is associated with drug-resistance of cancer cells and blocking autophagy could enhance the efficacy of chemotherapy [9–11]. Through degrading damaged organelles and misfolded proteins in autophagosomes, autophagy plays a vital role in maintaining the intracellular homeostasis and prolonging the survival of cancer cells under chemotherapeutic stress [12].

In the current study, we established doxorubicin (Dox)-resistant gallbladder cancer cells from the parental cells and screened the differentially expressed lncRNAs by transcriptome microarray analysis. Among the upregulated lncRNAs, we characterized and identified a novel lncRNA ENST00000425894 that is implicated in the chemoresistance of gallbladder cancer cells and termed it as gallbladder cancer drug resistance-associated lncRNA1 (GBCDRlnc1). In addition, GBCDRlnc1 is also significantly upregulated in gallbladder cancer tissues. The experiments in vitro and in vivo were conducted to assess the biological role of GBCDRlnc1 in gallbladder cancer. Through promoting the autophagic flux, GBCDRlnc1 induces the chemoresistance of gallbladder cancer cells. Mechanistically, mass spectrometry together with integrative analysis revealed that GBCDRlnc1 could interact with phosphoglycerate kinase 1 (PGK1) protein and regulate its stability by inhibiting its ubiquitination, eventually lead to the increased expression of ATG5-ATG12 conjugate.

## Materials and methods

### Cells and human tissues

The NOZ and GBC-SD human gallbladder cancer cell lines, purchased from the Health Science Research Resources Bank (Osaka, Japan) and the Cell Bank of the Chinese Academy of Science (Shanghai, China) were respectively maintained in Williams’ Medium E (Genom, China) and DMEM high glucose medium (HyClone, USA) supplemented with 10% fetal bovine serum (FBS, Gibco, USA). Cells were cultured at 37 °C in a humidified atmosphere of 5% CO_2_ and 95% air. The Dox (Meilunbio, China) resistant gallbladder cancer cells NOZ/Dox and GBC-SD/Dox were established in a stepwise manner by continuous exposing the cells to increasing concentration of Dox from 10 ng/ml to 1 μg/ml over a period of one year. The gemcitabine (GEM) and 5-fluorouracil (5-FU) used in this study were purchased from Meilunbio, China. The autophagy inhibitor chloroquine (CQ) and 3-Methyladenine (3-MA) were respectively purchased from Sigma, USA and Selleck, USA. The protein synthesis inhibitor cycloheximide (CHX) was purchased from Meilunbio, China. The proteasome inhibitor MG-132 was purchased from Beyotime, China.

Forty-five pairs of human gallbladder cancer tissues and their corresponding adjacent noncancerous tissues were obtained from gallbladder cancer patients who had undergone surgery in Xinhua Hospital (Shanghai JiaoTong University School of Medicine, Shanghai, China) and Eastern Hepatobiliary Surgery Hospital (Second Military Medical University, Shanghai, China) from November 2009 to October 2013. Written informed consent was obtained from all patients in accordance with the Declaration of Helsinki. The patients were diagnosed with gallbladder cancer according to histopathological evaluation, and no any pre-operative treatment was conducted. This study was approved by the Human Ethics Committee of Xinhua Hospital.

### Microarray analysis

Arraystar Human lncRNA Microarray V4.0 was used for this study. About 40,173 lncRNAs and 20,730 coding transcripts can be detected by the lncRNA microarray. Sample labeling and array hybridization were performed according to the Agilent One-Color Microarray-Based Gene Expression Analysis protocol (Agilent Technology, USA) with minor modifications. The hybridized arrays were scanned with using the Agilent DNA Microarray Scanner (No. G2505C). Agilent Feature Extraction software V11.0.1.1 was used to analyze acquired array images. Quantile normalization and subsequent data processing were performed with using the GeneSpring GX V12.1 software package (Agilent Technologies, USA). After quantile normalization of the raw data, lncRNAs and mRNAs that at least 3 out of 6 samples have flags in Present or Marginal (“All Targets Value”) were chosen for further data analysis. Differentially expressed lncRNAs and mRNAs with statistical significance between the two groups were identified through *P*-value/FDR filtering. Differentially expressed lncRNAs and mRNAs between the two samples were identified through Fold Change filtering. Differentially expressed genes were then identified through Fold Change as well as *P* value calculated with *t*-test. The threshold set for up- and down-regulated genes was a Fold Change > 2.0 and *P* value < 0.05. Hierarchical Clustering and combined analysis were performed using in-house scripts.

### RNA extraction and qRT-PCR

Total RNA was isolated from tissues or cell lines using Trizol reagent (Invitrogen, USA). RNA was reversed transcribed into cDNAs using the PrimeScript™ one step RT-PCR kit (TaKaRa, China) according to the manufacturer’s protocol. The mRNA level was measured using the SYBR® Premix DimmerEraser™ kit (TaKaRa, China) and the ABI7500 system (Applied Biosystems, USA). The relative mRNA expression change was calculated by using 2^-ΔΔCt^ method and the β-actin was used as an internal control for normalization. The primer sequences are listed in Additional file 1: Table S1.

### RNA interference and vectors

Small interfering RNAs (siRNAs) that specifically target human GBCDRlnc1 and PGK1 were purchased from GenePharma (Shanghai, China). The vectors pcDNA3.1-GBCDRlnc1 and pcDNA3.1-PGK1 were purchased from Sangon Biotech (Shanghai, China). Cells were cultured on six-well plates to confluency and transfected with siRNAs, vectors or negative control using Lipofectamine 2000 (Invitrogen, USA) according to the manufacturer’s protocol. The lentivirus vector containing the shRNA-GBCDRlnc1 was purchased from Genechem (Shanghai, China). Stably shRNA-GBCDRlnc1-transfected cells were selected by the treatment of puromycin (1 μg/ml, Solarbio, China). The RNA interference sequences are listed in Additional file 1: Table S1.

### In vitro and in vivo chemosensitivity assay

For in vitro experiments, the drug-resistant or parental gallbladder cancer cells with or without transfection were seed into 96-well plates (3 × 10^3^ cells/well), and the medium containing different concentrations of drugs (Dox or GEM) with or without the autophagy inhibitor (CQ or 3-MA) was added. After incubation for 48 h, the absorbance (450 nm) was assessed by the water-soluble tetrazolium salt assay using the Cell Counting Kit-8 (CCK-8, Dojindo, Japan) according to the manufacturer’s protocol. Then we drew the cell growth curve and calculated the 50% inhibition of growth (IC_50_) value of each cell line to each drug.

For in vivo experiments, NOZ/Dox cells (100 μl, approximately 1.0 × 10^7^ cells) were subcutaneously injected into each 4-week-old male nude mouse. CQ (60 mg/kg) and Dox (1 mg/kg) were intraperitoneally injected into mice every three days. The mice were monitored daily, and the tumor volumes were assessed (0.5 × length × width^2^) per week. After five weeks, mice were sacrificed, and all tumor grafts were excised, photographed. To explore the function of GBCDRlnc1 in vivo, NOZ/Dox and GBC-SD/Dox cells (100 μl, approximately 1.0 × 10^7^ cells) that stably transfected with Lv-shRNA-GBCDRlnc1 or Lv-control were subcutaneously injected into mice. The dosage of Dox was same as above. All tumor grafts and forty-five human gallbladder cancer tissues were subjected to immunohistochemical staining. The antibodies for immunohistochemical staining was ATG5 (1:100, Proteintech, China), ATG12 (1:100, Proteintech, China), LC3 (1:200, Novus, USA), p62 (1:100, Proteintech, China) and PGK1 (1:200, Proteintech, China). The expression levels of PGK1 in forty-five human gallbladder cancer tissues were evaluated according to the methods described by Shao et al. [13]. All animal experiments were performed in animal laboratory center of Xinhua Hospital of Shanghai JiaoTong University School of Medicine (Shanghai, China). The study protocol was approved by the Animal Care and Use committee of Xinhua Hospital.

### RNA pull-down and mass spectrometry analysis

The resultant plasmid DNA was linearized with restriction enzyme NotI. Biotin-labeled RNAs were in vitro transcribed with the Biotin RNA Labeling Mix (Roche, USA) and T7 RNA polymerase (Roche, USA), treated with RNase-free DNase I (Roche, USA) and purified with the RNeasy Mini Kit (Qiagen, Germany). Cell extract (2 μg) was mixed with biotinylated RNA (100 pmol). Washed Streptavidin agarose beads (100 ml) were added to each binding reaction and further incubated at room temperature for 1 h. Beads were washed briefly three times and boiled in SDS buffer. The retrieved protein was separated using electrophoresis then silver-stained and analyzed by mass spectrometry. The retrieved protein PGK1 was further validated by standard western blot.

### RNA immunoprecipitation (RIP)

RIP assay was performed using the EZ-Magna RIP RNA-Binding Protein Immunoprecipitation Kit (Millipore, USA) as described previously [6]. Cells were lysed in complete RIP lysis buffer and cleared lysates were then incubated with RIP buffer containing magnetic beads conjugated to human anti-PGK1 antibody (Proteintech, China). The negative control was normal mouse IgG (Beyotime, China), and the positive control was SNRNP70 (Millipore, USA). The coprecipitated RNAs were isolated by Trizol reagent (Invitrogen, USA) and detected by qRT-PCR. The gene-specific primer sequences used for the qRT-PCR of the RIP assay were listed in Additional file 1: Table S1.

### Co-immunoprecipitation (co-IP) assay

Co-IP assay was performed using Pierce Co-Immunoprecipitation Kit (Thermo, USA) according to the manufacturer’s protocol. The ubiquitin antibody used for Co-IP assay was purchased from Proteintech, China.

### Cell proliferation and cell cycle analysis

Cell proliferation was assessed using CCK-8 (Dojindo, Japan) and colony formation assay as previously described [14, 15]. For CCK-8 assay, cells were seeded into 96-well plates (1 × 10^3^ cells/well) and the absorbance (450 nm) was measured every 24 h for 96 h. For colony formation assay, cells were seeded into 6-well plates (1 × 10^3^ cells/well) and cultured in media with 10% FBS. After two weeks, cells were treated with methanol and stained with 0.1% crystal violet. The number of visible colonies was counted. Cell cycle was analyzed by flow cytometry on a FACS Calibur (BD Biosciences, USA) as previously described [15]. Cells were fixed by pre-cold 70% ethanol for 12–24 h at 4 °C and incubated in staining solution (5 U/mL RNaseA and 10 μg/mL propidium iodide) for 30 min at 37 °C. The flow cytometer was used for assessment.

### Transwell assay

Transwell assay was performed by using 24-well plates with 8-μm chamber inserts (Corning Life Science, USA) as previously described [15]. The inserts were precoated with Matrigel (50 μl/well, BD, USA) and 2 × 10^5^ cells were seeded in the upper chamber with serum free medium in triplicate. Medium containing 10% FBS was added to the lower chamber as chemo-attractant. After incubation for 24 h, the cells above the Matrigel layer were removed by cotton swab, and the cells below the membrane were fixed by methanol, stained with 0.1% crystal violet for 10 min, and counted from five randomly chosen fields for each well.

### Western blot

Western blot was performed as previously described [15]. The antibodies for the western blot were ATG3 (1:1000, Cell Signaling Technology, USA), ATG5 (1:1000, Cell Signaling Technology, USA), ATG7 (1:1000, Cell Signaling Technology, USA), ATG12 (1:1000, Cell Signaling Technology, USA), Beclin1 (1:1000, Proteintech, China), LC3 (1:1000, Novus, USA), p62 (1:1000, Proteintech, China), PGK1 (1:1000, Proteintech, China), Ubiquitin (1:1000, Proteintech, China) and β-actin (1:5000, Proteintech, China).

### Stably expressing stubRFP-sensGFP-LC3

The lentivirus vector containing the stubRFP-sensGFP-LC3 reporter was purchased from Genechem (Shanghai, China). Stably expressing stubRFP-sensGFP-LC3 cells were selected by the treatment of puromycin (1 μg/ml). After different treatment, the cells were fixed and then analyzed using fluorescence microscopy (Olympus BX51, Japan).

### 3′ rapid amplification of cDNA ends (RACE)

3′-RACE was performed to determine the transcriptional termination site of GBCDRlnc1 using a SMARTer™ RACE cDNA Amplification Kit (Clontech, USA) according to the manufacturer’s protocol. The gene-specific primer sequences used for the qRT-PCR of the RACE assay were listed in Additional file 1: Table S1.

### Subcellular fractionation

To determine the cellular localization of GBCDRlnc1, we isolated and collected the cytoplasm and nuclear fractions of gallbladder cancer cells by using RNeasy Midi Kit (Qiagen, Germany) as previously described [14]. RNAs extracted from each of the fractions were subjected to following qRT-PCR analysis of the levels of GBCDRlnc1, GAPDH and U6. The primer sequences of GAPDH and U6 were listed in Additional file 1: Table S1.

### Transmission electron microscopy (TEM)

Cells were fixed for 2 h in 2% glutaraldehyde containing 0.1 mol/l phosphate-buffered saline at 4 °C, incubated in 1% osmium tetroxide containing 0.1 mol/l phosphate-buffered saline for 2 h at 4 °C, dehydrated in graded ethanol, saturated in graded Epikote and embedded, cut into 50-nm ultrathin sections, stained with lead citrate and finally viewed using Philip CM-120 TEM (Philips, Netherlands).

### Statistical analysis

Data were presented as mean ± standard deviation (SD). Paired samples *t*-test was used to analyze the expression differences of GBCDRlnc1 between gallbladder cancer tissues and neighboring noncancerous tissues. Independent samples *t*-test was used to analyze the differences between groups. Kaplan-Meier method was used to analyze the survival, and log-rank test was used to determine the significance. *P* values were two-side and a *P* value less than 0.05 was considered to be statistically significant.

## Results

### The enhancement of autophagic activity of dox-resistant gallbladder cancer cells contributes to their drug-resistant property

**Fig. 1:**
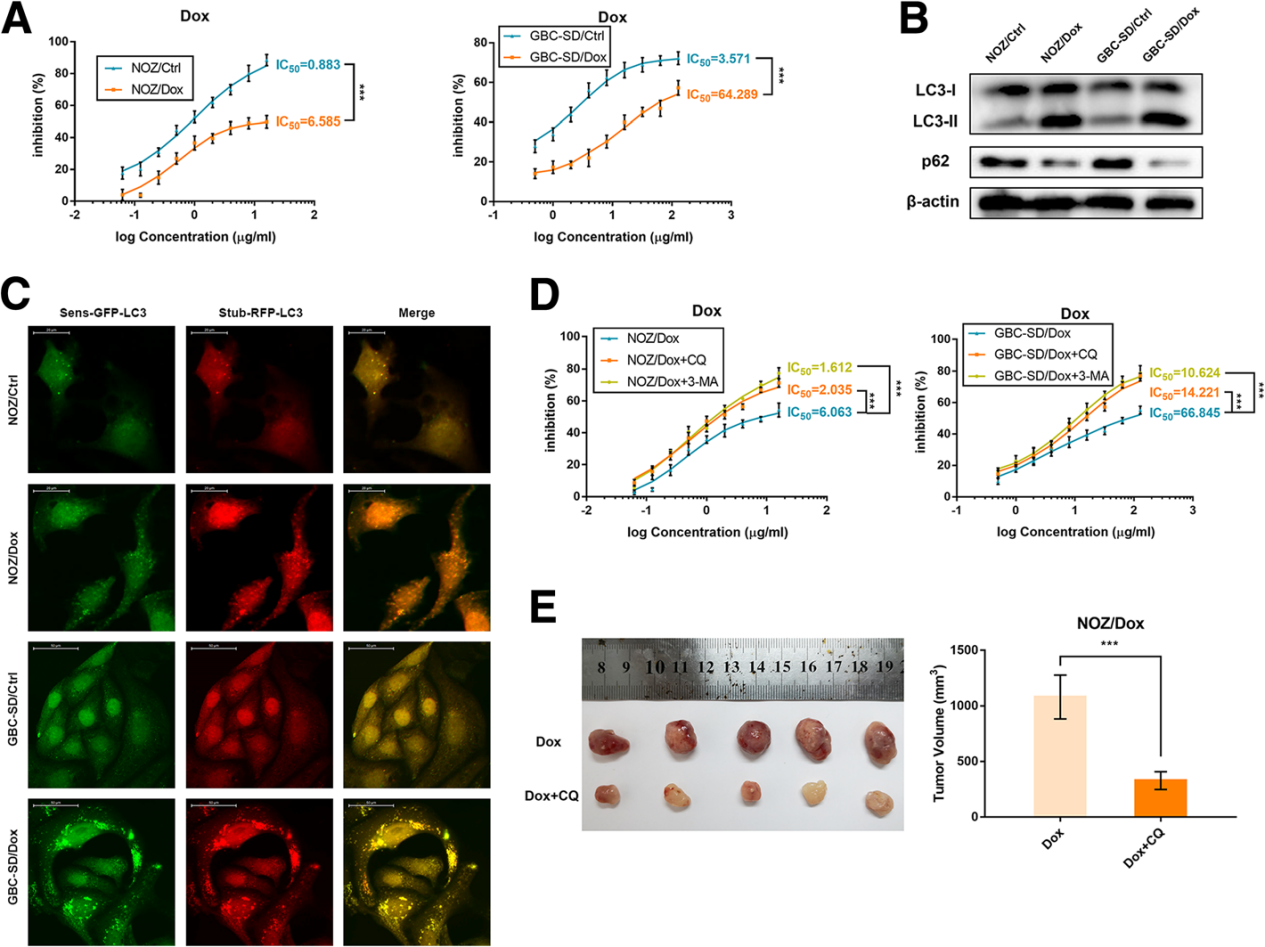
The drug-resistant property of Dox-resistant gallbladder cancer cells is associated with their enhanced autophagic activity. **a** The sensitivities of Dox-resistant gallbladder cancer cells and their parental cells with Dox were determined by CCK-8 assay. **b** The protein levels of LC3 and p62 in Dox-resistant gallbladder cancer cells and their parental cells were determined by western blot assay. **c** Dox-resistant gallbladder cancer cells and their parental cells that stably express the stubRFP-sensGFP-LC3 fusion protein were established and observed by the fluorescence microscope. **d** The sensitivities of Dox-resistant gallbladder cancer cells and their parental cells under different treatments with Dox were determined by CCK-8 assay. **e** The nude mice carrying tumors from NOZ/Dox under Dox with or without CQ were shown. Average tumor volume for each group was calculated. The mean ± SD of triplicate experiments were plotted, ***P < 0.001

To reveal the molecular mechanisms that contribute to the drug-resistance of gallbladder cancer cells, we firstly established the Dox-resistant gallbladder cancer cells and confirmed the IC_50_ values for Dox in NOZ/Dox and GBC-SD/Dox cells were both significantly higher than their parental cells (Fig. 1a). Next, we sought if there were some differences in cell proliferation between drug-resistant cells and their parental cells. However, we did not observe any significant change between groups (Additional file 2: Figure S1). Through allowing the residual or metastatic cancer cells to tolerate the cytotoxic stress, autophagy confers them the property of drug-resistance [16]. Thus, we hypothesized that drug-resistant gallbladder cancer cells might exhibit increased autophagy. To our knowledge, LC3-II and p62 are autophagic markers. Compared with their parental cells, NOZ/Dox and GBC-SD/Dox cells both presented enhanced conversion from LC3-I to LC3-II and increased p62 degradation (Fig. 1b). To validate the enhanced autophagic flux in drug-resistant cells, we established stably expressing stubRFP-sensGFP-LC3 gallbladder cancer cells using a lentiviral vector to localize and assessed the autophagic flux. The sensGFP is sensitive to the PH change owing to the fusion of autophagosomes and lysosomes, whereas stubRFP is stable. Consistently with the results above, we observed the enhanced autophagosomal-lysosomal fusion process in NOZ/Dox and GBC-SD/Dox cells using a fluorescence microscope (Fig. 1c). For in vitro experiments, the IC_50_ values for Dox in NOZ/Dox and GBC-SD/Dox cells were successfully reversed by treatment of the autophagy inhibitor CQ or 3-MA (Fig. 1d). Furthermore, with the combination of Dox and CQ, tumor was significantly smaller compared with animals treated with Dox alone (Fig. 1e). Collectively, these data demonstrated that the drug-resistant property of gallbladder cancer cells is related to their enhanced autophagic activity and inhibition of autophagy is capable to enhance the cytotoxicity of Dox in gallbladder cancer cells in vitro and in vivo.

### LncRNAs expression profile in dox-resistant gallbladder cancer cells

**Fig. 2:**
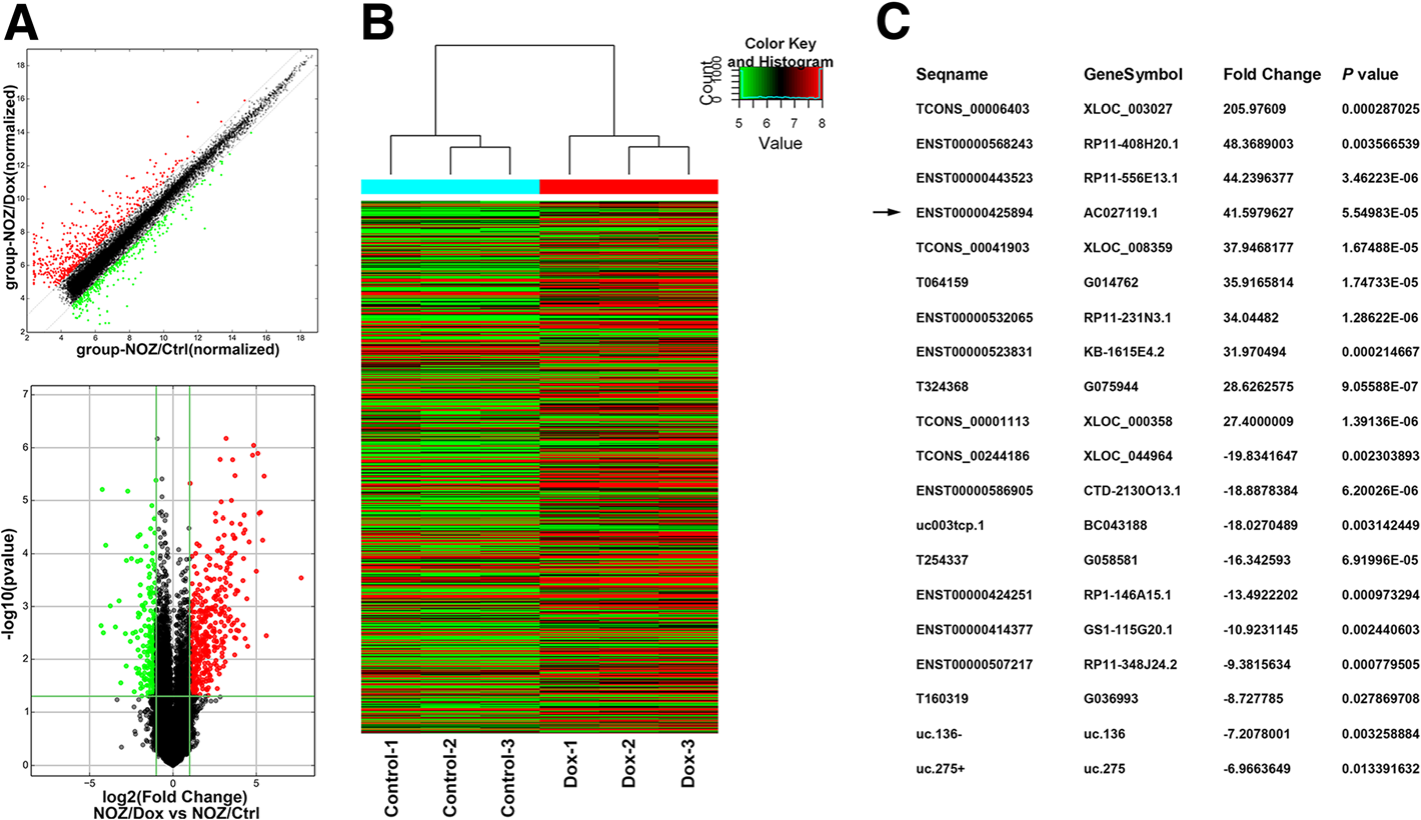
LncRNA expression profile in NOZ/Dox cells. **a** The scatter plot was used for assessing the variation in lncRNA expression between NOZ/Dox and NOZ/Ctrl cells. The values of x and y axes in the scatter plot were the normalized signal values of the samples (log2 scaled). The gray dotted lines are fold-change lines. The lncRNAs above the top gray dotted line and below the bottom gray dotted line indicated more than 2.0-fold change of lncRNAs between the two compared samples. The volcano plot was constructed using fold-change values and *P*-values. The vertical lines correspond to 2.0-fold up and down, respectively, and the horizontal line represents a *P*-value of 0.05. The green and red point in the plot represents the differentially expressed lncRNAs with statistical significance. **b** The cluster heat map showed the differentially expressed lncRNAs over 2.0-fold change. Red colour indicates high expression level, and green colour indicates low expression level. **c** The top 10 upregulated lncRNAs and top 10 downregulated lncRNAs. The arrow indicates GBCDRlnc1

To identify the transcripts that potentially induce gallbladder cancer cells to resist Dox in vitro, we compared the expression profiles of lncRNAs and mRNAs in NOZ/Dox and its parental cells NOZ/Ctrl by human microarray analysis. The scatter and volcano plots showed the variation of lncRNAs expression between NOZ/Dox and NOZ/Ctrl (Fig. 2a). In total, 457 upregulated lncRNAs and 266 downregulated lncRNAs with Fold Change > 2.0 were identified (Additional file 3: Table S2 and Additional file 4: Table S3). The hierarchical clustering analysis showed the differentially expressed lncRNAs (Fig. 2b). And we listed the top 10 upregulated lncRNAs and top 10 downregulated lncRNAs in Fig. 2c. To validate the microarray results, we randomly selected 5 upregulated lncRNAs, 5 downregulated lncRNAs, 3 upregulated mRNAs and 3 down-regulated mRNAs and assessed their relative expression in NOZ/Dox and NOZ/Ctrl (Additional file 5: Figure S2). According to the results of qRT-PCR, we concluded that the microarray findings are reliable, and revealed that a cluster of lncRNAs are aberrantly expressed in Dox-resistant gallbladder cancer cells.

Among the aberrantly high expressed lncRNAs, we aimed at screening a lncRNA that relates to the enhanced autophagic activity and contributes to the drug-resistant property of Dox-resistant gallbladder cancer cells. We selected the top 5 ranked candidates with significant *P* value (*P* < 0.001) and knocked down their expression respectively. In these 5 lncRNAs, we found that only the lncRNA GBCDRlnc1 (ENST00000425894, AC027119.1, GENCODE) is associated to the enhanced autophagic activity and drug-resistant property of Dox-resistant gallbladder cancer cells (data not shown). According to UCSC genome database (http://genome.ucsc.edu/), we found that GBCDRlnc1 is located at human chromosome 3: 6004529–6,165,807 and the genomic length of GBCDRlnc1 is 467 bp. The sequence of full-length GBCDRlnc1 is presented in Additional file 6: Figure S3A. The sequencing of 3′-RACE-PCR products revealed the boundary between the universal anchor primer and GBCDRlnc1 (Additional file 6: Figure S3B). Through prediction in online databases (Coding Potential Assessment Tool, http://lilab.research.bcm.edu/cpat/), we found that GBCDRlnc1 is indeed a noncoding RNA (Additional file 6: Figure S3C).

### GBCDRlnc1 is upregulated in gallbladder cancer tissues and correlates with the prognosis of gallbladder cancer patients

**Fig. 3:**
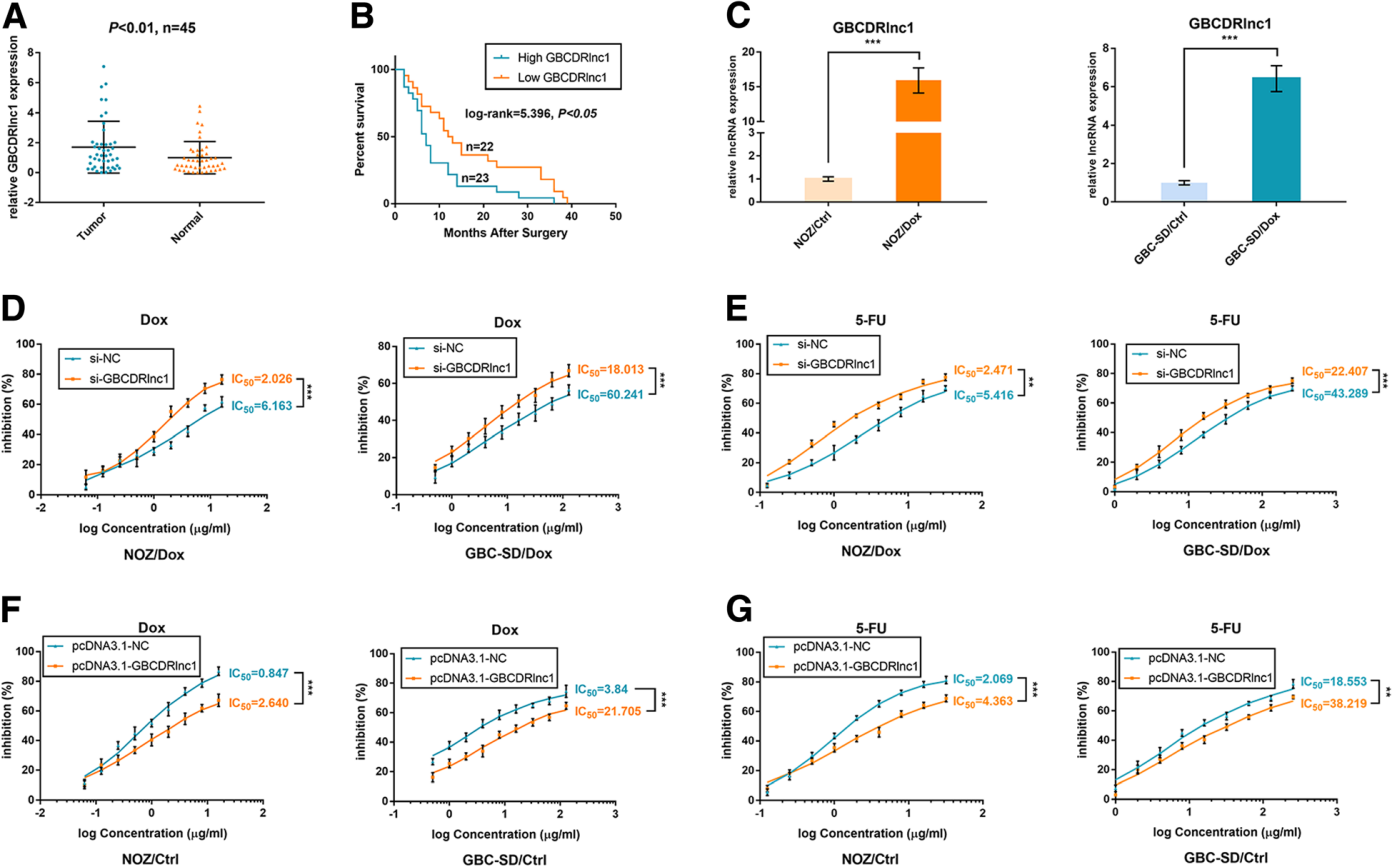
GBCDRlnc1 is upregulated in gallbladder cancer tissues and promotes chemoresistance of gallbladder cancer cells in vitro. **a** Relative expression of GBCDRlnc1 in gallbladder cancer tissues and neighboring noncancerous tissues was detected by qRT-PCR (*P* < 0.01, *n* = 45). **b** Kaplan–Meier method with the log-rank test was used to analyze the overall survival curves of patients in high and low GBCDRlnc1 expression groups (log-rank = 5.396, *P* < 0.05). **c** Relative expression of GBCDRlnc1 in Dox-resistant gallbladder cancer cells and their parental cells was determined by qRT-PCR. **d** The sensitivities of Dox-resistant gallbladder cancer cells under different transfection with Dox were determined by CCK-8 assay. **e** The sensitivities of Dox-resistant gallbladder cancer cells under different transfection with 5-FU were determined by CCK-8 assay. **f** The sensitivities of the parental gallbladder cancer cells under different transfection with Dox were determined by CCK-8 assay. **g** The sensitivities of the parental gallbladder cancer cells under different transfection with 5-FU were determined by CCK-8 assay. The mean ± SD of triplicate experiments were plotted, **P < 0.01, ***P < 0.001

To explore the role of GBCDRlnc1 in gallbladder cancer, we performed qRT-PCR to detect the transcript levels of GBCDRlnc1 in forty-five pairs of gallbladder cancer tissues and their corresponding adjacent noncancerous tissues. As shown in Fig. 3a, the transcript levels of GBCDRlnc1 are significantly higher in gallbladder cancer tissues compared with their corresponding adjacent noncancerous tissues, after normalizing to β-actin. To explore the correlation between the transcript levels of GBCDRlnc1 and the prognosis of gallbladder cancer patients, we divided these gallbladder cancer patients into high- and low-GBCDRlnc1 group according to the median ratio of relative GBCDRlnc1 expression in gallbladder cancer tissues. Kaplan-Meier analysis suggested that patients with high-GBCDRlnc1 expression levels have a significantly shorter overall survival than those with low-GBCDRlnc1 expression levels (Fig. 3b). Based on the clinical and pathologic characteristics of patients, we performed statistical analysis and found that the expression level of GBCDRlnc1 is significantly associated with histological grade and TNM stage (Table 1).

**Table 1:** The association of GBCDRlnc1 expression in forty-five gallbladder cancer patients with clinicopathologic charateristics

		GBCDRlnc1 expression		
Characteristics	Case number	High (*n* = 23)	Low (*n* = 22)	*P*-value
Gender				0.235
Male	14	9	5	
Female	31	14	17	
Age				0.641
≤ 60	25	12	13	
> 60	20	11	9	
Tumor size				0.299
≤ 5 cm	17	7	10	
> 5 cm	28	16	12	
Local invasion				0.138
Yes	28	17	11	
No	17	6	10	
Lymph node metastasis				0.300
Yes	24	14	10	
No	21	9	12	
Histological grade				0.035*
well and morderately	30	12	18	
Poorly and others	15	11	4	
TNM stage				0.023*
I-II	17	5	12	
III-IV	28	18	10	

*P< 0.05

### GBCDRlnc1 induces autophagy-associated chemoresistance of gallbladder cancer cells in vitro

**Fig. 4:**
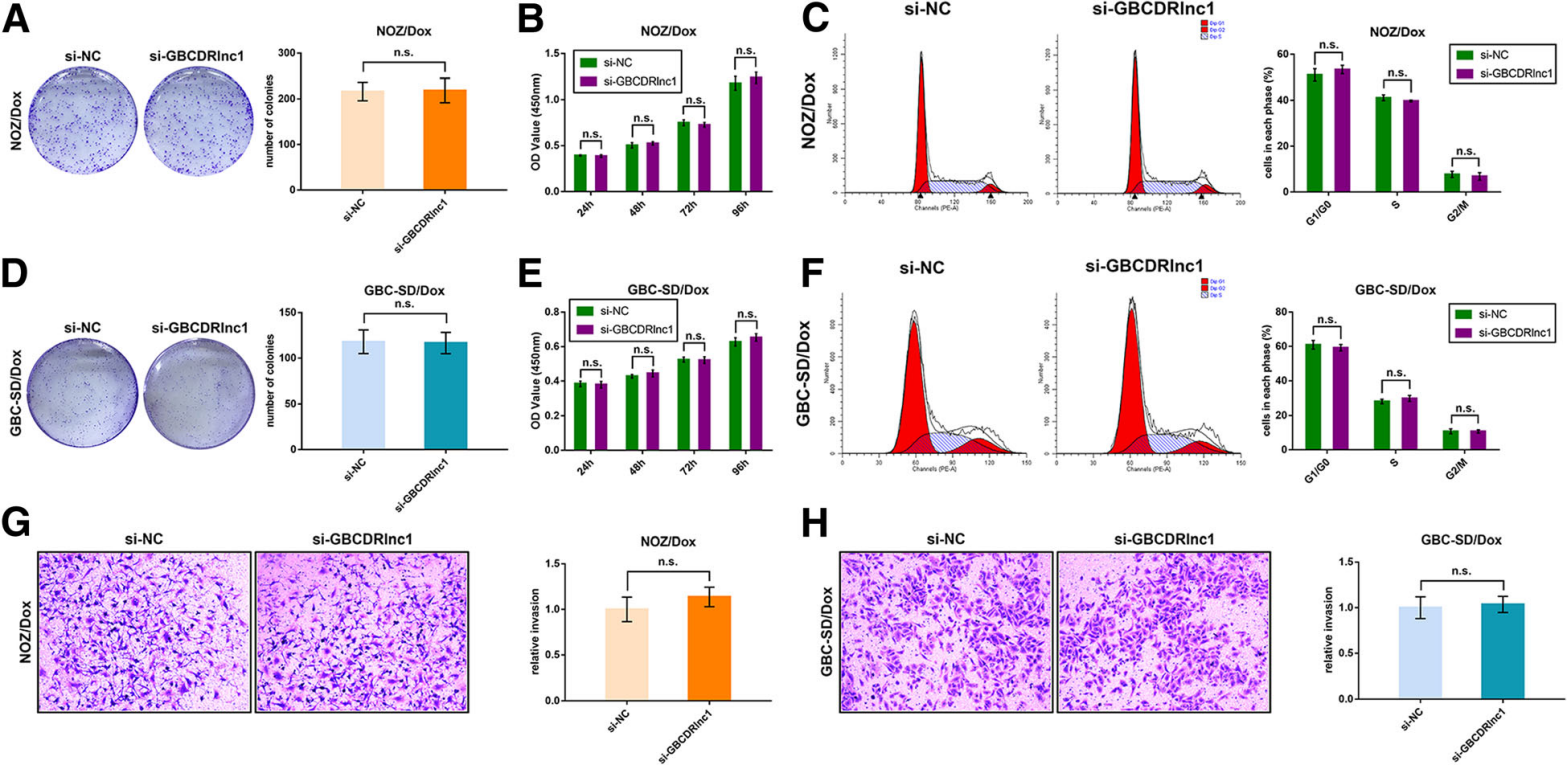
Knockdown of GBCDRlnc1 does not affect the proliferation and invasion of Dox-resistant gallbladder cancer cells in vitro. **a** The coloning ability of NOZ/Dox cells under different transfection was determined by colony formation assay. **b** The cell viability of NOZ/Dox cells under different transfection was determined by CCK8 assay. **c** Flow cytometric analyses were performed to determine the cell cycle progression in NOZ/Dox cells under different transfection. **d** The coloning ability of GBC-SD/Dox cells under different transfection was determined by colony formation assay. **e** The cell viability of GBC-SD/Dox cells under different transfection was determined by CCK8 assay. **f** Flow cytometric analyses were performed to determine the cell cycle progression in GBC-SD/Dox cells under different transfection. **g**-**h** The cell invasion ability of Dox-resistant gallbladder cancer cells under different transfection was determined by transwell assay. The mean ± SD of triplicate experiments were plotted, n.s., not statistically significant

First, we performed qRT-PCR to detect the expression levels of GBCDRlnc1 in Dox-resistant gallbladder cancer cells and their parental cells. Compared with NOZ/Ctrl and GBC-SD/Ctrl cells, GBCDRlnc1 was significantly upregulated in NOZ/Dox and GBC-SD/Dox cells (Fig. 3c). To further explore the role of GBCDRlnc1 in gallbladder cancer, we transfected NOZ/Dox and GBC-SD/Dox cells that presented relatively high expression levels of GBCDRlnc1 with two individual siRNAs against GBCDRlnc1. Then we selected si-GBCDRlnc1–1 for the subsequent experiments for its higher inhibition efficiency (Additional file 7: Figure S4A). Plasmids containing pcDNA3.1-GBCDRlnc1 were transfected into NOZ/Ctrl and GBC-SD/Ctrl cells that presented relatively low expression levels of GBCDRlnc1 to upregulated the expression of GBCDRlnc1, and the overexpression efficiency was validated by qRT-PCR (Additional file 7: Figure S4B). As shown in Fig. 3d-e and Additional file 7: Figure S4C, GBCDRlnc1 knockdown significantly enhanced the sensitivity of NOZ/Dox and GBC-SD/Dox cells to Dox, 5-FU and GEM. While, GBCDRlnc1 overexpression heightened the IC_50_ values of NOZ/Ctrl and GBC-SD/Ctrl cells to the three chemotherapeutic agents (Fig. 3f-g and Additional file 7: Figure S4D). However, GBCDRlnc1 knockdown did not change the proliferation, cell cycle distribution and invasion of drug-resistant gallbladder cancer cells (Fig. 4).

**Fig. 5:**
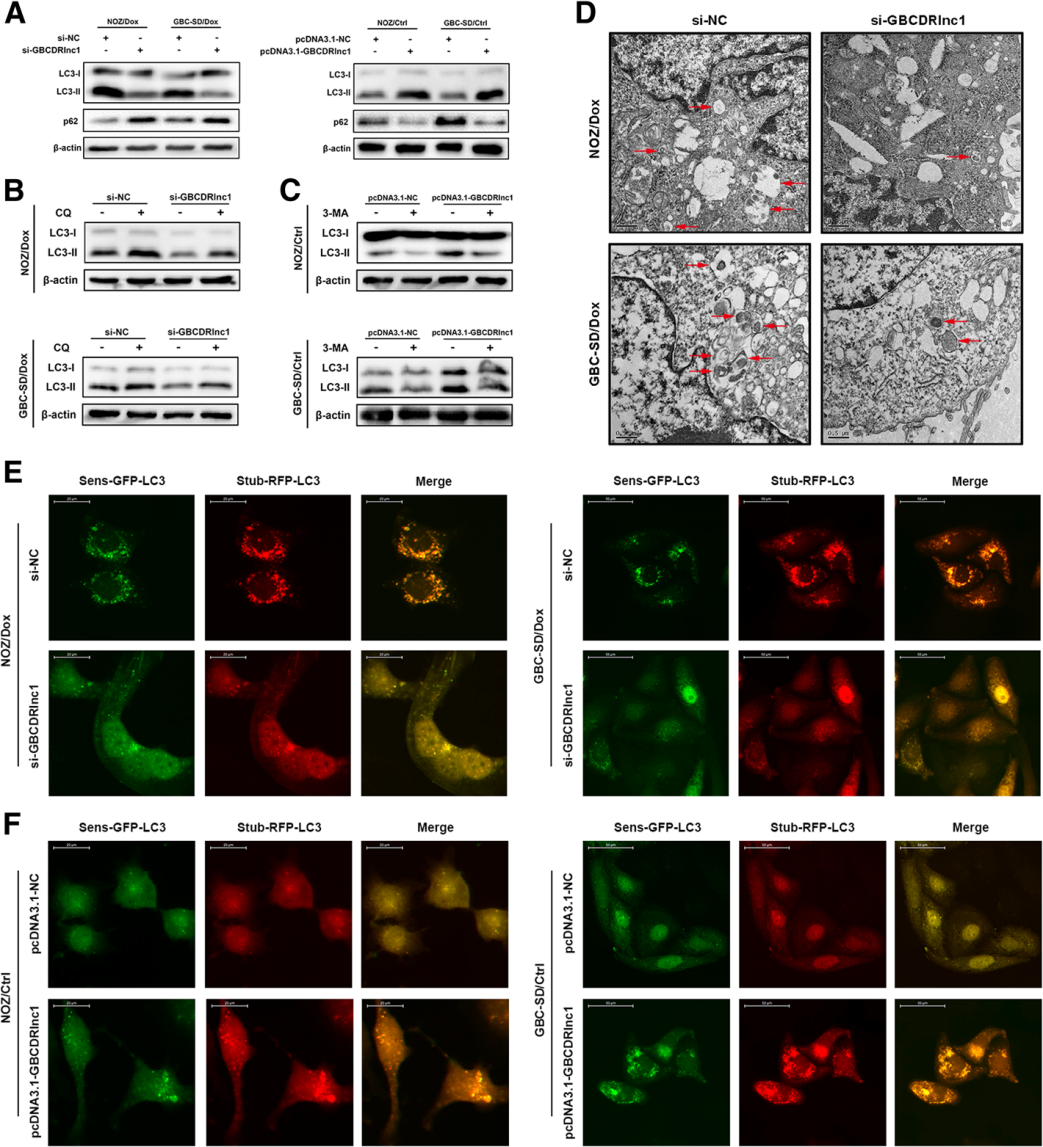
GBCDRlnc1 promotes autophagy of gallbladder cancer cells in vitro. **a** The protein levels of LC3 and p62 in Dox-resistant gallbladder cancer cells and their parental cells under different transfection were determined by western blot assay. **b** The protein levels of LC3 in Dox-resistant gallbladder cancer cells under different transfection with CQ (10 μM) were determined by western blot assay. **c** The protein levels of LC3 in the parental gallbladder cancer cells under different transfection with 3-MA (10 mM) were determined by western blot assay. **d** Autophagy was evaluated in Dox-resistant gallbladder cancer cells under different transfection using TEM. **e** Dox-resistant gallbladder cancer cells stably expressing stubRFP-sensGFP-LC3 under different transfection were observed by the fluorescence microscope. **f** The parental gallbladder cancer cells stably expressing stubRFP-sensGFP-LC3 under different transfection were observed by the fluorescence microscope

Then we explored whether GBCDRlnc1 regulates the chemosensitivity of gallbladder cancer cells via inducing autophagy. The results of western blot showed that the conversion from LC3-I to LC3-II was inhibited while the expression of p62 was elevated in si-GBCDRlnc1-transfected Dox-resistant gallbladder cancer cells, and the opposite phenomenon was observed in GBCDRlnc1-overexpression NOZ/Ctrl and GBC-SD/Ctrl cells (Fig. 5a). Furthermore, autophagic flux was also detected by assessing the conversion from LC3-I to LC3-II in the presence or absence of CQ and 3-MA. CQ and 3-MA are two autophagy inhibitors, the former raises the intralysosomal pH and suppresses the fusion of autophagosome and lysosome at late stage, the latter inactivates class III phosphatidylinositol 3-kinases and blocks autophagosome formation at early stage [10]. The results of western blot showed that the expression levels of LC3-II were elevated by CQ, while the combination of si-GBCDRlnc1 and CQ reduced the expression levels of LC3-II in drug-resistant gallbladder cancer cells compared with the combination of si-NC and CQ (Fig. 5b). The treatment of 3-MA in pcDNA3.1-GBCDRlnc1-transfected NOZ and GBC-SD cells reversed the increased expression levels of LC3-II by pcDNA3.1-GBCDRlnc1 alone (Fig. 5c). In addition, we used TEM to evaluate autophagosomes and detected a substantial decrease in the accumulation of autophagic vesicles in si-GBCDRlnc1-transfected Dox-resistant gallbladder cancer cells (Fig. 5d). Through a fluorescence microscope, we observed that both the numbers of stubRFP-sensGFP-LC3 puncta and stubRFP-LC3 puncta were paralleled with the expression levels of GBCDRlnc1 (Fig. 5e-f). These results suggested that GBCDRlnc1 is capable of inducing autophagy at initial stage and promoting the chemoresistance of gallbladder cancer cells in vitro.

### GBCDRlnc1 directly interacts with PGK1 and upregulates its protein level via inhibiting PGK1 ubiquitination in gallbladder cancer cells in vitro

**Fig. 6:**
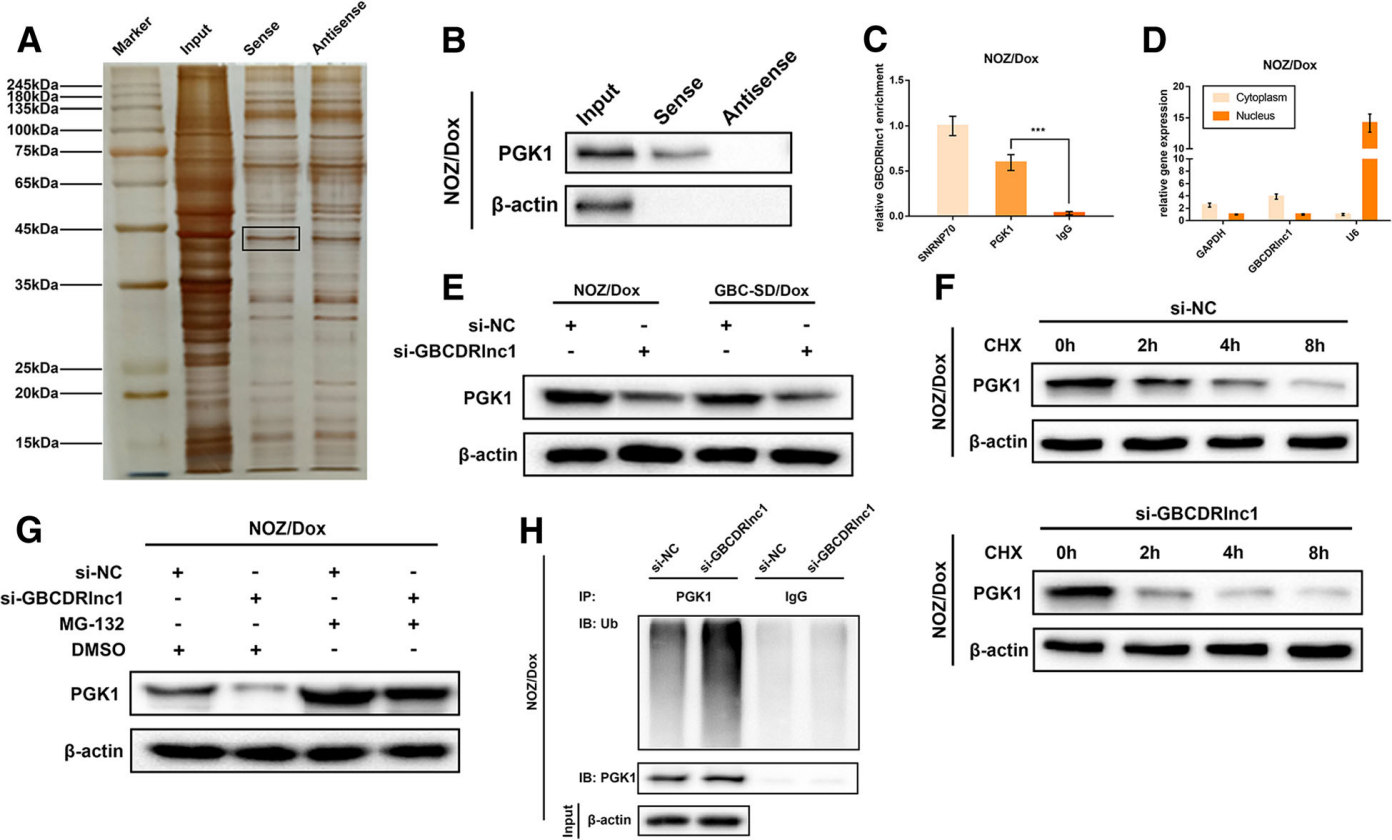
GBCDRlnc1 directly interacts with PGK1 and upregulated its protein level via inhibiting PGK1 ubiquitination in gallbladder cancer cells in vitro*.* **a** Silver-stained SDS-PAGE gel of proteins immunoprecipitated from NOZ/Dox cell extract by the sense and antisense RNA of GBCDRlnc1. The two lanes were used for mass spectrum determination by the liquid chromatography dual mass spectrometry method. The frame indicates PGK1. **b** RNA pull-down assay was conducted using biotin-labeled GBCDRlnc1 probe and determined the PGK1 expression by western blot assay. Antisense of the GBCDRlnc1 probe was used as negative control. **c** Amount of GBCDRlnc1 bound to SNRNP70 (a positive control), PGK1 or IgG (a negative control) was detected by qRT-PCR after RIP in NOZ/Dox cells. **d** Relative expression of GBCDRlnc1 in cell cytoplasm or nucleus of NOZ/Dox cells was determined by qRT-PCR. **e** The protein levels of PGK1 in Dox-resistant gallbladder cancer cells under different transfection were determined by western blot assay. **f** The protein levels of PGK1 in NOZ/Dox cells under different transfection with CHX (20 mg/ml) were determined by western blot assay. **g** The protein levels of PGK1 in NOZ/Dox cells under different transfection with MG-132 (5 μM) were determined by western blot assay. **h** NOZ/Dox cells under different transfection were treated with MG-132 (5 μM) for 24 h. Cell lysates were immunoprecipitated with antibodies against PGK1 or IgG. The levels of ubiquitination were analysed by western blot. Bottom, input from cell lysates. The mean ± SD of triplicate experiments were plotted, ***P < 0.001

To elucidate the underlying molecular mechanism by which GBCDRlnc1 promoted autophagy in gallbladder cancer cells, we performed RNA pull-down assay to identify the protein partners of GBCDRlnc1 in NOZ/Dox cells. The RNA-associated proteins that were pulled down by biotin-labeled sense- or antisense-GBCDRlnc1 were analyzed by silver staining (Fig. 6a). Then the two protein bands were subjected to mass spectrometry and we found that PGK1 was uniquely expressed in the GBCDRlnc1 pull-down group (Additional file 8: Table S4). It has been well-known that PGK1 catalyzes a crucial step of glycolysis and plays a key role in glycolytic ATP production [17]. Qian et al. reported that the protein kinase activity of PGK1 is implicated in the activation of autophagy [18, 19]. We performed immunoblotting and further validated that GBCDRlnc1 could interact with PGK1 directly (Fig. 6b), vice versa, RIP assay indicated that the antibody to PGK1 could capture significantly greater enrichment of GBCDRlnc1 than the antibody to IgG in NOZ/Dox and GBC-SD/Dox cells (Fig. 6c and Additional file 9: Figure S5A). These results suggested that GBCDRlnc1 specifically interacts with PGK1 in gallbladder cancer cells.

To explore the intracellular localization of GBCDRlnc1 in drug-resistant gallbladder cancer cells, first, we used the online prediction databases (lncLocator, www.csbio.sjtu.edu.cn/bioinf/lncLocator) and found that GBCDRlnc1 is most possibly located in cytoplasm (Additional file 9: Figure S5B). Next, we performed the subcellular fractionation assay and validated the aforementioned results (Fig. 6d and Additional file 9: Figure S5C). To explore the effects of GBCDRlnc1 on PGK1, we manipulated the expression levels of GBCDRlnc1 in gallbladder cancer cells and detected the alteration of PGK1 mRNA and protein levels. As shown in Additional file 9: Figure S5D, the mRNA levels of PGK1 were not significantly changed when GBCDRlnc1 was silenced or overexpressed in gallbladder cancer cells. However, the protein levels of PGK1 were reduced in NOZ/Dox and GBC-SD/Dox cells when GBCDRlnc1 was silenced and upregulated in NOZ/Ctrl and GBC-SD/Ctrl cells when GBCDRlnc1 was overexpressed (Fig. 6e and Additional file 9: Figure S5E). Based on these results, we speculated that the synthesis and/or degradation of PGK1 protein might be regulated by GBCDRlnc1. To this end, we treated NOZ/Dox cells with the protein synthesis inhibitor CHX and found that knockdown of GBCDRlnc1 dramatically shortened the half-life of PGK1 (Fig. 6f and Additional file 9: Figure S5F). Moreover, treatment of si-GBCDRlnc1 transfected NOZ/Dox cells with the proteasome inhibitor MG-132 heightened the expression levels of PGK1 (Fig. 6g). While we observed similar phenomenon in GBC-SD/Dox cells (Additional file 9: Figure S5G). These results demonstrated that GBCDRlnc1 inhibits the proteasome-dependent degradation of PGK1 in drug-resistant gallbladder cancer cells. To further validate the ubiquitin-proteasome pathway was responsible for the GBCDRlnc1-mediated degradation of PGK1, we performed Co-IP assay to detect the ubiquitination of PGK1. As expected, the ubiquitination of PGK1 in NOZ/Dox and GBC-SD/Dox cells was dramatically heightened by GBCDRlnc1 knockdown (Fig. 6h and Additional file 9: Figure S5H). Given these, these results suggested that the direct interaction between GBCDRlnc1 and PGK1 prevents the ubiquitination and degradation of PGK1 in gallbladder cancer cells in vitro.

### Knockdown of PGK1 suppresses autophagy-associated chemoresistance of gallbladder cancer cells in vitro

**Fig. 7:**
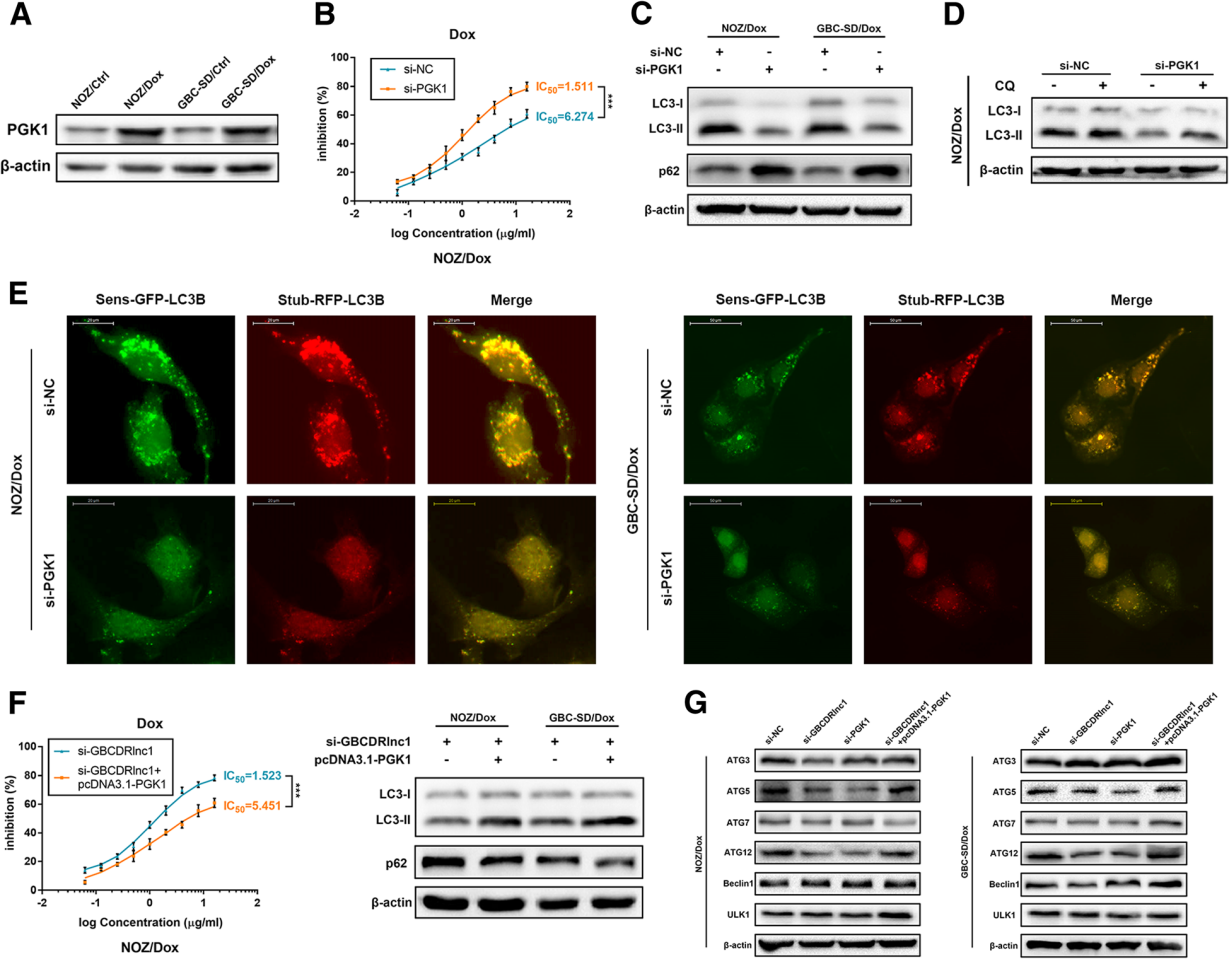
Knockdown of PGK1 suppresses autophagy-associated chemoresistance of gallbladder cancer cells in vitro*.* **a** The protein levels of PGK1 in Dox-resistant gallbladder cancer cells and their parental cells were determined by western blot assay. **b** The sensitivities of NOZ/Dox cells under different transfection with Dox were determined by CCK-8 assay. **c** The protein levels of LC3 and p62 in Dox-resistant gallbladder cancer cells under different transfection were determined by western blot assay. **d** The protein levels of LC3 in NOZ/Dox cells under different transfection with CQ (10 μM) were determined by western blot assay. **e** Dox-resistant gallbladder cancer cells stably expressing stubRFP-sensGFP-LC3 under different transfection were observed by the fluorescence microscope. **f** The sensitivities of NOZ/Dox cells under different transfection with Dox were determined by CCK-8 assay. The protein levels of LC3 and p62 in Dox-resistant gallbladder cancer cells under different transfection were determined by western blot assay. **g** The protein levels of ATG3, ATG5, ATG7, ATG12 and Beclin1 in Dox-resistant gallbladder cancer cells under different transfection were determined by western blot assay. The mean ± SD of triplicate experiments were plotted, ***P < 0.001

To explore whether PGK1 is involved in gallbladder cancer cells chemoresistance, we performed western blot to examine the expression levels of PGK1 in Dox-resistant gallbladder cancer cells and their parental cells. The results showed that the protein levels of PGK1 in NOZ/Dox and GBC-SD/Dox cells were significantly higher than that in NOZ/Ctrl and GBC-SD/Ctrl, which further validated the effect of GBCDRlnc1 on PGK1 (Fig. 7a). To silence the expression of PGK1 in drug-resistant gallbladder cancer cells, we designed two PGK1-specific siRNAs and chose the si-PGK1–2 for PGK1 knockdown (Additional file 10: Figure S6A). When endogenous PGK1 was effectively silenced, the IC_50_ values of NOZ/Dox and GBC-SD/Dox cells to Dox were significantly reduced (Fig. 7b and Additional file 10: Figure S6B). Moreover, the results of western blot and stubRFP-sensGFP-LC3 staining indicated that PGK1 knockdown could repress the autophagic flux of drug-resistant gallbladder cancer cells at initial stage (Fig. 7c-e and Additional file 10: Figure S6C). To upregulate the expression of PGK1, we transfected gallbladder cancer cells with plasmids containing pcDNA3.1-PGK1 and validated the efficiency of overexpression by western blot (Additional file 10: Figure S6D). The rescue experiments indicated that PGK1 overexpression abrogated the inhibitory effect of GBCDRlnc1 knockdown on the chemoresistance and autophagy of gallbladder cancer cells (Fig. 7f and Additional file 10: Figure S6E). Thus, we concluded that the function of GBCDRlnc1 on the chemoresistance and autophagic activity of gallbladder cancer cells is through its positive mediation of PGK1 in vitro.

In addition, we selected several genes (ATG3, ATG5, ATG7, ATG12, Beclin1, ULK1) that were primarily involved in the progression of autophagy from phagophore initiation to autolysosome fusion, and postulated that they might be related to the autophagy repression at initial stage owing to GBCDRlnc1 knockdown. Interestingly, the results of western blot showed that only the protein levels of ATG5 and ATG12 were both significantly reduced in GBCDRlnc1 or PGK1 knockdown gallbladder cancer drug-resistant cells, while PGK1 overexpression rescued the inhibitory effect of GBCDRlnc1 knockdown on ATG5 and ATG12 expression (Fig. 7g). While, genetic and in vitro studies suggested that ATG5-ATG12 conjugate is an essential complex for the autophagophore elongation [20, 21]. There data indicated that ATG5-ATG12 conjugate might be a downstream target of GBCDRlnc1/PGK1 pathway in vitro.

### Knockdown of GBCDRlnc1 inhibits autophagy and improves the sensitivity of gallbladder cancer cells to dox in vivo

**Fig. 8:**
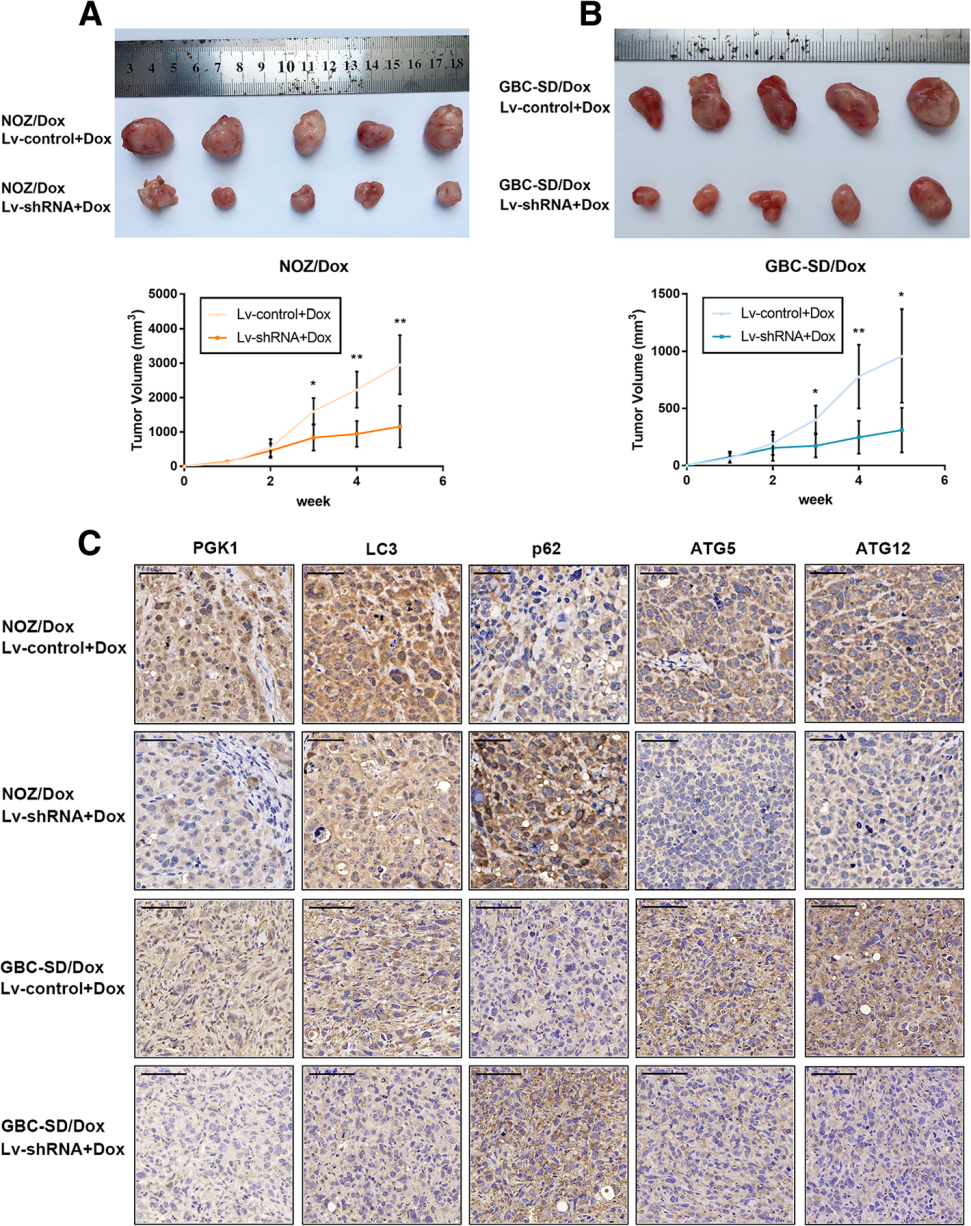
Knockdown of GBCDRlnc1 inhibits autophagy and improves the sensitivity of gallbladder cancer cells to Dox in vivo*.* **a** The nude mice carrying tumors from NOZ/Dox under different transfection with Dox were shown. Tumor growth curves were calculated per week. **b** The nude mice carrying tumors from GBC-SD/Dox under different transfection with Dox were shown. Tumor growth curves were calculated per week. **c** The PGK1, LC3, p62, ATG5 and ATG12 expression and positive cell numbers was determined by immunohistochemical staining. Scale bar = 50 μm (NOZ/Dox) or 100 μm (GBC-SD/Dox). The mean ± SD of triplicate experiments were plotted, *P < 0.05, **P < 0.01, ***P < 0.001

**Fig. 9:**
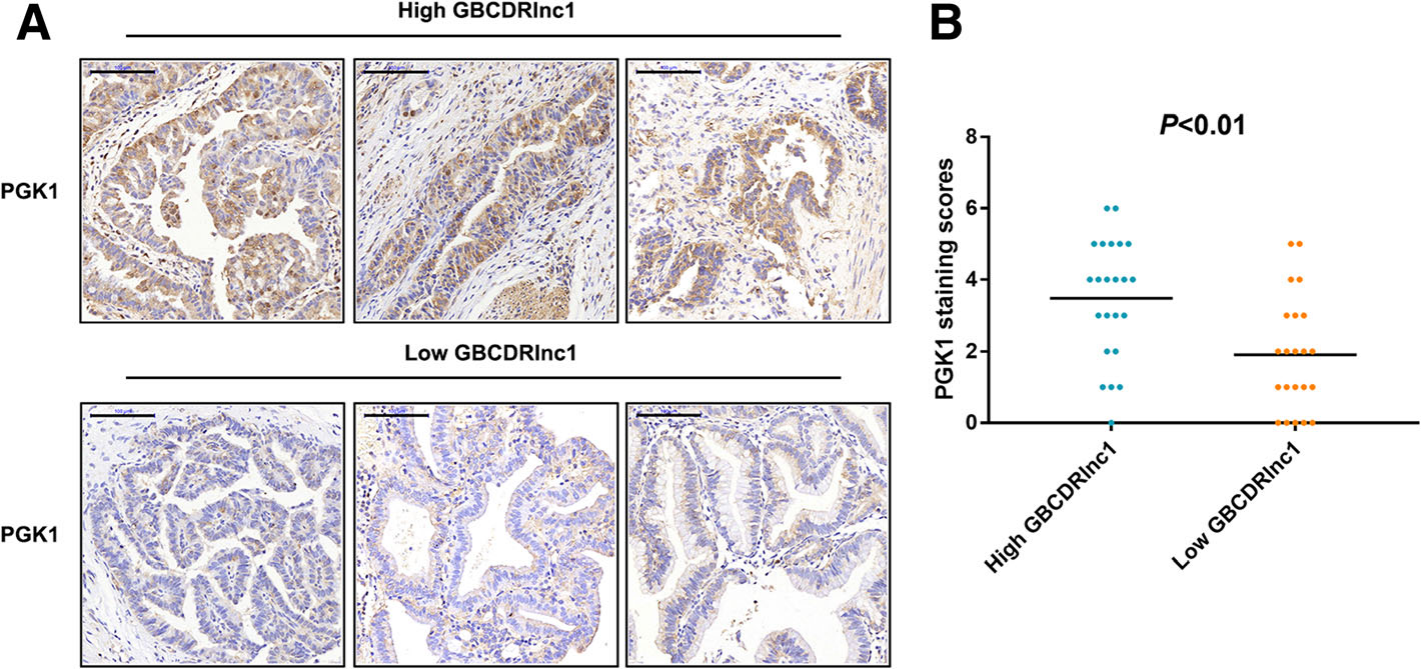
The correlation between the expression level of GBCDRlnc1 and PGK1 in human gallbladder cancer tissues is positive*.* **a** Representative immunohistochemical staining micrographs showing PGK1 expression in the divided high- and low-GBCDRlnc1 human gallbladder cancer tissues. Scale bar = 100 μm. **b** Scatterplots of the average staining scores of PGK1 expression in the divided high- and low-GBCDRlnc1 human gallbladder cancer tissues (*P* < 0.01)

To explore the function of GBCDRlnc1 on Dox-resistant gallbladder cancer cells in vivo, NOZ/Dox and GBC-SD/Dox cells with stable transfection with Lv-shRNA-GBCDRlnc1 or Lv-control were subcutaneously injected into nude mice. Dox was intraperitoneally injected into mice every three days. With the treatment of Dox, tumor developed significantly faster in Lv-control group than that in Lv-shRNA-GBCDRlnc1 group (Fig. 8a-b). While, we performed qRT-PCR in mouse tumor tissues and confirmed the significance of GBCDRlnc1 knockdown in Lv-shRNA-GBCDRlnc1 group (Additional file 10: Figure S6F). Furthermore, immunohistochemical staining of PGK1, LC3, p62, ATG5 and ATG12 showed that PGK1, ATG5 and ATG12 were dramatically downregulated in Lv-shRNA-GBCDRlnc1 group and confirmed that knockdown of GBCDRlnc1 inhibited the autophagic activity of gallbladder cancer cells in vivo, which is consistent with the aforementioned results in vitro (Fig. 8c). To further evaluate the positive correlation between GBCDRlnc1 and PGK1, we performed immunohistochemical staining to examined the expression level of PGK1 in the divided high- and low-GBCDRlnc1 human gallbladder cancer tissues. As shown in Fig. 9a, the tissues with high GBCDRlnc1 expression had stronger PGK1 staining than the tissues with low GBCDRlnc1 expression. And the average staining score for PGK1 expression was significantly higher in high GBCDRlnc1 group (Fig. 9b).

## Discussion

Dox, an anthracycline antibiotic with broad-spectrum anticancer activity, is one of the mainstay chemotherapeutic drugs for clinical treatment of various cancers, including gallbladder cancer [22]. Nonetheless, the low chemotherapy response rate in gallbladder cancer (less than 30%) and severe adverse events (particularly cardiotoxicity) limits its clinical use [23, 24]. Chemotherapy resistance is mainly attributed to the adaptation of cancer cell itself to the multiple stresses induced by chemotherapeutic drugs, which is related to a variety of mechanisms, such as angiogenesis, inactivation of apoptosis, induction of autophagy, drug efflux and metabolism, increased repair of damage, etc. [25, 26]. Autophagy is an evolutionarily conserved catabolic process, which is constitutively activated in chemotherapy [27]. Current evidence supported the notion that manipulating the activity of autophagy might be a useful therapeutic strategy to enhance the chemosensitivty of some anticancer agents, including Dox [28, 29].

In the present study, we found that there is no difference in gallbladder cancer cell proliferation between drug-resistant cells and their parental cells, but the drug-resistant cells presented increased autophagic activity without the stimulation of Dox. Notably, co-treatment with CQ or 3-MA to inhibit autophagy in drug-resistant gallbladder cancer cells enhanced their sensitivity to Dox in vitro and in vivo. CQ is an established drug that has been widely used for malaria prophylaxis, while its potential use as a tumor chemosensitizer due to its inhibitory of autophagy has been investigated in recent years [30]. Shi et al. reported that the combination of sorafenib with CQ heightens the effectiveness of tumor suppression in hepatocellular carcinoma [31]. Similar with our results, Qiu et al. reported that the cooperation of CQ with Dox improves the cytotoxicity in Dox-resistant human breast cancer cells [32]. As is well-known, the toxicity of CQ is relatively low, the pharmacological properties of CQ is well understood and the price of CQ is quite low [33]. These characteristics make it suitable as a tumor chemosensitizer and the present study formed the basis for future clinical trials in advanced gallbladder cancer patients.

To our knowledge, we were the first to established the drug-resistant gallbladder cancer cells and explored the mechanism through which these cells presented increased autophagic activity. We identified a small number of aberrantly expressed lncRNAs and mRNAs in Dox-resistant gallbladder cancer cells compared with the parental cells and found a new lncRNA transcript ENST00000425894 (GBCDRlnc1), which is significantly upregulated in Dox-resistant gallbladder cancer cells. Furthermore, we confirmed that GBCDRlnc1 is also significantly upregulated in gallbladder cancer tissues in a cohort of forty-five gallbladder cancer patients, and the high expression of GBCDRlnc1 is associated with histological grade and advanced TNM stage. Higher expression of GBCDRlnc1 tends to have poorer histological grade, hinting that high expression of GBCDRlnc1 might mediate the malignant differentiation of gallbladder cancer cells. GBCDRlnc1 expression is further significantly upregulated in gallbladder cancer patients with advanced TNM stage, which suggested that GBCDRlnc1 might play an important role in gallbladder cancer progression. The survival analysis determined that GBCDRlnc1 overexpression is related to shorter survival time of gallbladder cancer patients. These data indicated that GBCDRlnc1 could be a potential prognostic indicator for gallbladder cancer. However, the association of GBCDRlnc1 expression and clinical chemotherapeutic characteristics of gallbladder cancer patients should be confirmed in future studies with a large cohort of samples.

Consistently, we confirmed that GBCDRlnc1 induces chemoresistance of gallbladder cancer cells via a mechanism involving autophagy in vitro and in vivo by loss- and gain-of function assays. However, we found that GBCDRlnc1 has no effect on the proliferation, cell cycle distribution and invasion of drug-resistant gallbladder cancer cells. Mechanically, we identified that the oncogenic function of GBCDRlnc1 in gallbladder cancer is associated with the interaction of this lncRNA and PGK1. By preventing its ubiquitin-mediated degradation, GBCDRlnc1 posttranslationally regulates the expression level of PGK1. LncRNAs could play a role in multiple pathways via interacting with specific miRNAs, mRNAs and proteins [34]. Fatica et al. reported that lncRNA GAS5 directly binds to the DNA binding domain of glucocorticoid receptor, thus blocks them from interacting with the DNA response elements [35]. Zhang et al. reported that lncRNA HOTAIR binds to the androgen receptor to block its interaction with the E3 ubiquitin ligase MDM2, thus inhibits its ubiquitination [36]. Moreover, we found that lncRNA MetaLnc9 could interact with PGK1 and relieve its ubiquitination in non-small lung cancer [37]. Given this, we supposed that GBCDRlnc1 might act as a “molecular decoy” in gallbladder cancer cells, which directly binds to PGK1 and interferes the interaction of PGK1 and ubiquitin. Consequently, the results of RNA pull-down, RIP and Co-IP assays together supported this hypothesis. Nevertheless, the underlying mechanisms of how GBCDRlnc1 mediates the ubiquitination associated enzymes should be further investigation.

PGK1, a key enzyme in glycolytic pathway, catalyzes the transfer of 1,3-diphosphoglycerate to 3-phosphoglycerate and ADP to ATP [38]. Previous studies reported that PGK1 is significantly upregulated in various malignant tumors, such as gastric carcinoma [39], hepatocellular carcinoma [40] and pancreatic ductal adenocarcinoma [41]. While, there have been several lines of evidence indicating that PGK1 overexpression could induce the chemoresistance of breast and ovarian cancer cells [42, 43]. In this study, we found that PGK1 abrogates GBCDRlnc1-mediated autophagy and chemoresistance in drug-resistant gallbladder cancer cells, hinting that GBCDRlnc1 functions in a PGK1-dependent manner. The well-defined formation of autophagosomes involves four steps: initiation, phagophore nucleation, autophagosome elongation and closure, and autolysosome fusion [44]. Furthermore, we confirmed that PGK1 knockdown represses the autophagic flux of drug-resistant gallbladder cancer cells at initial stage, which is in accord with the effect of GBCDRlnc1 knockdown on autophagy. Additionally, it was reported that PGK1 K388 acetylation mediates the phosphorylation of Beclin1 S30, thus affects the initiation of autophagy [18]. However, we did not detect whether PGK1 activity could change upon GBCDRlnc1 expression alteration in this study, and further investigation for this is necessary. Through the lysine at residue 149, ATG5 is conjugated to the carboxy-terminal glycine of ATG12 to form an irreversible covalent complex, which controls the initiation and elongation steps of autophagic vacuole formation [21]. Furthermore, it is widely accepted that ATG5 and ATG12 are promoters in chemoresistance or radioresistance [45–48]. Our study showed that the protein levels of ATG5 and ATG12 were both decreased when GBCDRlnc1 or PGK1 was downregulated in Dox-resistant gallbladder cancer cells and the effect could be neutralized by PGK1 overexpression, which suggested that ATG5-ATG12 conjugate might be a downstream target of GBCDRlnc1/PGK1 pathway.

In summary, we identified that lncRNA GBCDRlnc1 induces autophagy and drug-resistance of gallbladder cancer cells by interacting with PGK1 and preventing its degradation, which eventually upregulates ATG5-ATG12 expression. Therefore, GBCDRlnc1/PGK1/ATG5-ATG12 conjugate signaling pathway might be a novel therapeutic target for gallbladder cancer chemotherapy.

## Additional files

Additional file 1: **Table S1.** Information of the qRT-PCR primer, siRNA and shRNA sequence. (XLSX 174 kb)

Additional file 2: **Figure S1.** There is no difference in cell proliferation between Dox-resistant gallbladder cancer cells and their parental cells. (A) The coloning ability of NOZ/Dox and NOZ/Ctrl cells was determined by colony formation assay. (B) The cell viability of NOZ/Dox and NOZ/Ctrl cells was determined by CCK8 assay. (C) The coloning ability of GBC-SD/Dox and GBC-SD/Ctrl cells was determined by colony formation assay. (D) The cell viability of GBC-SD /Dox and GBC-SD /Ctrl cells was determined by CCK8 assay. The mean ± SD of triplicate experiments were plotted, n.s., not statistically significant. (TIF 5491 kb)

Additional file 3: **Table S2.** 457 upregulated lncRNAs in NOZ/Dox with Fold Change > 2.0. (XLSX 45 kb)

Additional file 4: **Table S3.** 266 downregulated lncRNAs in NOZ/Dox with Fold Change > 2.0. (XLSX 29 kb)

Additional file 5: **Figure S2.** Expression levels of 10 lncRNAs (A-B) and 6 mRNAs (C-D) by qRT-PCR in NOZ/Dox and NOZ/Ctrl cells. The mean ± SD of triplicate experiments were plotted, **P < 0.01, ***P < 0.001. (TIF 7922 kb)

Additional file 6: **Figure S3.** The information of GBCDRlnc1. (A) Nucleotide sequence of the full-length human GBCDRlnc1 gene. (B) The full-length GBCDRlnc1 sequence (highlighted) cloned from 3′-RACE. (C) The coding potential assessment tool showed that GBCDRlnc1 lacks protein-coding potential (the coding probability more than 0.364 is deemed to be able to code protein). (TIF 10162 kb)

Additional file 7: **Figure S4.** The efficiency of GBCDRlnc1 expression regulation of gallbladder cancer cells in vitro. (A) Relative expression of GBCDRlnc1 in Dox-resistant gallbladder cancer cells with si-GBCDRlnc1 was determined by qRT-PCR. (B) Relative expression of GBCDRlnc1 in Dox-resistant gallbladder cancer cells with pcDNA3.1-GBCDRlnc1 was determined by qRT-PCR. (C) The sensitivities of Dox-resistant gallbladder cancer cells under different transfection with GEM were determined by CCK-8 assay. (D) The sensitivities of the parental gallbladder cancer cells under different transfection with GEM were determined by CCK-8 assay. The mean ± SD of triplicate experiments were plotted, *P < 0.05, **P < 0.01, ***P < 0.001. (TIF 4499 kb)

Additional file 8: **Table S4.** List of possible GBCDRlnc1-sence and GBCDRlnc1-antisence interacting proteins identified by mass spectrometry. (XLSX 34 kb)

Additional file 9: **Figure S5.** GBCDRlnc1 inhibits PGK1 ubiquitination in gallbladder cancer cells in vitro*.* (A) Amount of GBCDRlnc1 bound to SNRNP70 (a positive control), PGK1 or IgG (a negative control) was determined by qRT-PCR after RIP in GBC-SD/Dox cells. (B) The online software lncLocator was used to predict the location of GBCDRlnc1. (C) Relative expression of GBCDRlnc1 in cell cytoplasm or nucleus of GBC-SD/Dox cells was determined by qRT-PCR. (D) Relative expression of PGK1 in Dox-resistant gallbladder cancer cells under different transfection was determined by qRT-PCR. (E) The protein levels of PGK1 in the parental gallbladder cancer cells under different transfection were determined by western blot assay. (F) The protein levels of PGK1 in GBC-SD/Dox cells under different transfection with CHX (20 mg/ml) were determined by western blot assay. (G) The protein levels of PGK1 in GBC-SD/Dox cells under different transfection with MG-132 (5 μM) were determined by western blot assay. (H) GBC-SD/Dox cells under different transfection were treated with MG-132 (5 μM) for 24 h. Cell lysates were immunoprecipitated with antibodies against PGK1 or IgG. The levels of ubiquitination were analysed by western blot. Bottom, input from cell lysates. The mean ± SD of triplicate experiments were plotted, ***P < 0.001, n.s., not statistically significant. (TIF 6627 kb)

Additional file 10: **Figure S6.** Knockdown of PGK1 suppresses autophagy-associated chemoresistance of gallbladder cancer cells in vitro*.* (A) The protein levels of PGK1 in Dox-resistant gallbladder cancer cells under different transfection were determined by western blot assay. (B) The sensitivities of GBC-SD/Dox cells under different transfection with Dox were determined by CCK-8 assay. (C) The protein levels of LC3 in GBC-SD/Dox cells under different transfection with CQ (10 μM) were determined by western blot assay. (D) The protein levels of PGK1 in Dox-resistant gallbladder cancer cells under different transfection were determined by western blot assay. (E) The sensitivities of GBC-SD/Dox cells under different transfection with Dox were determined by CCK-8 assay. (F) Relative expression of GBCDRlnc1 in mouse tumor tissues under different transfection with Dox was determined by qRT-PCR. The mean ± SD of triplicate experiments were plotted, ***P < 0.001. (TIF 4358 kb)

## Abbreviations

3-MA: 3-Methyladenine
5-FU: 5-fluorouracil
CCK-8: Cell Counting Kit-8
CHX: Cycloheximide
Co-IP: Co-Immunoprecipitation
CQ: Chloroquine
Dox: Doxorubicin
FBS: Fetal bovine serum
GBCDRlnc1: drug resistance-associated lncRNA1
GEM: Gemcitabine
IC_50_: 50% inhibition of growth
lncRNA: long non-coding RNA
PGK1: Phosphoglycerate kinase 1
RACE: Rapid amplification of cDNA ends
RIP: RNA immunoprecipitation
SD: Standard deviation
siRNA: small interfering RNAs
TEM: Transmission electron microscopy
