# Correction: Long non-coding RNA GBCDRlnc1 induces chemoresistance of gallbladder cancer cells by activating autophagy

**DOI:** 10.1186/s12943-022-01691-w

**Published:** 2022-12-13

**Authors:** Qiang Cai, Shouhua Wang, Longyang Jin, Mingzhe Weng, Di Zhou, Jiandong Wang, Zhaohui Tang, Zhiwei Quan

**Affiliations:** 1 Department of General Surgery, XinHua Hospital, Shanghai JiaoTong University School of Medicine, Shanghai, 200092 China; 2 Department of Surgery, Shanghai Institute of Digestive Surgery, Ruijin Hospital, Shanghai JiaoTong University School of Medicine, Shanghai, 200025 China

**Correction: Mol Cancer 18, 82 (2019)**

**https://doi.org/10.1186/s12943-019-1016-0**

In the originally published version of this article [1], after receiving the comment, we have carefully re-checked the raw data and confirmed the error in Fig. 8*C. *We inadvertently used an image for p62 in ‘GBC-SD/Dox Lv-shRNA+Dox’ from the original images for ATG5 in ‘GBC-SD/Dox Lv-control+Dox’ as we assembled Fig. 8*C. *We confirm that the mistake would not affect the results or conclusions of the paper. The image of p62 in ‘GBC-SD/Dox Lv-shRNA+Dox’ has now been replaced with the correct one.

**Fig. 8:**
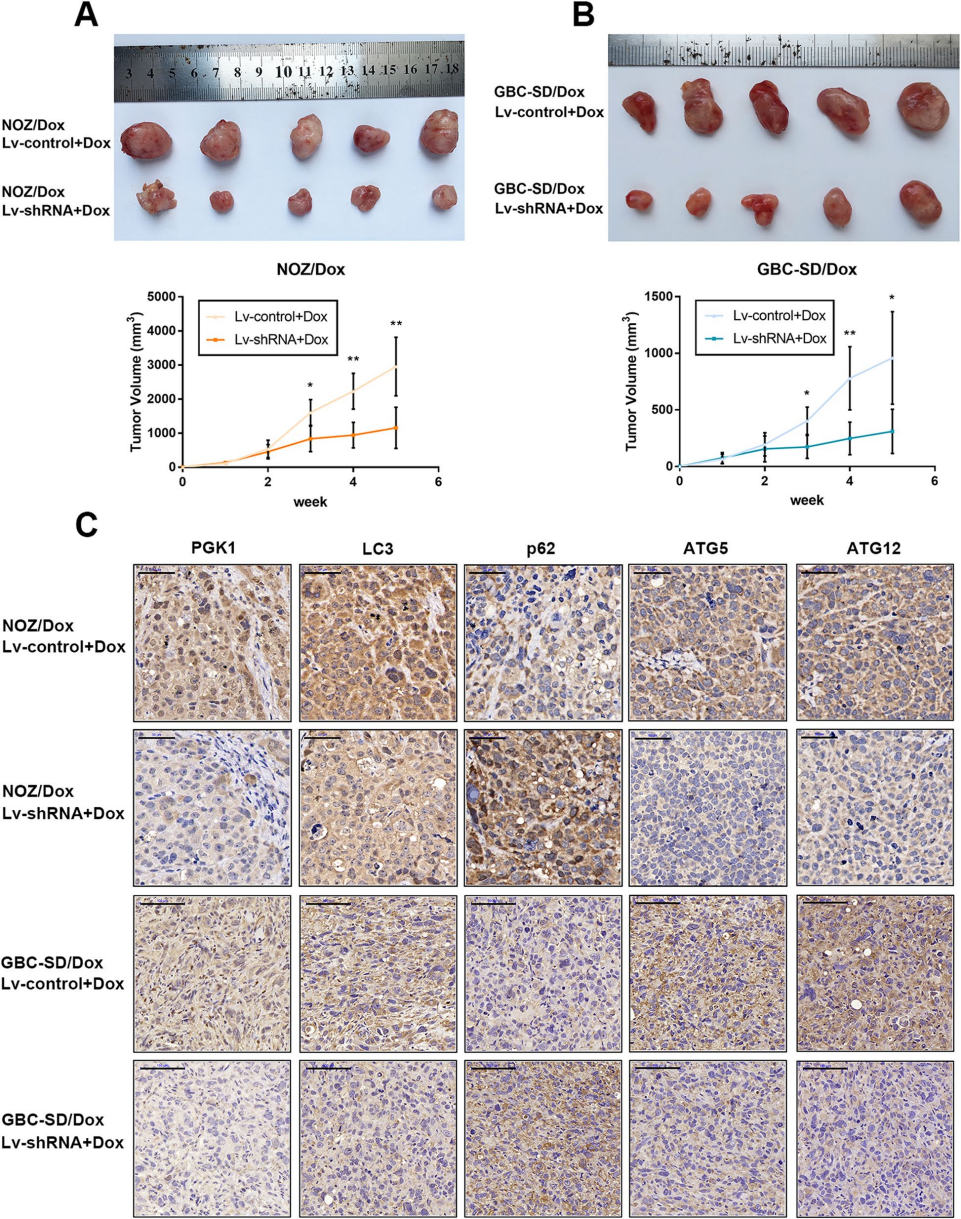
Knockdown of GBCDRlnc1 inhibits autophagy and improves the sensitivity of gallbladder cancer cells to Dox in vivo. **a** The nude micecarrying tumors from NOZ/Dox under different transfection with Dox were shown. Tumor growth curves were calculated per week. **b** The nude mice carrying tumors from GBC-SD/Dox under different transfection with Dox were shown. Tumor growth curves were calculated per week. **c** The PGK1, LC3, p62, A TG5 and ATG12 expression and positive cell numbers was determined by immunohistochemical staining. Scale bar = 50 μm (NOZ/Dox) or 100 μm (GBC-SD/Dox). The mean ± SD of triplicate experiments were plotted, *P < 0.05, **P < 0.01, ***P < 0.001
