# Correction: Long non-coding RNA GBCDRlnc1 induces chemoresistance of gallbladder cancer cells by activating autophagy

**DOI:** 10.1186/s12943-023-01760-8

**Published:** 2023-03-17

**Authors:** Qiang Cai, Shouhua Wang, Longyang Jin, Mingzhe Weng, Di Zhou, Jiandong Wang, Zhaohui Tang, Zhiwei Quan

**Affiliations:** 1 Department of General Surgery, XinHua Hospital, Shanghai JiaoTong University School of Medicine, Shanghai, 200092 China; 2 Department of Surgery, Shanghai Institute of Digestive Surgery, Ruijin Hospital, Shanghai JiaoTong University School of Medicine, Shanghai, 200025 China

**Correction** **:** **Mol Cancer 18, 82 (2019)**

**https://doi.org/10.1186/s12943-019-1016-0**

After receiving the comment, we have carefully re-checked the raw data and confirmed the error in Fig. 4A. The two images of ‘si-NC of NOZ/Dox’ in Fig. 4A in this article and the ‘si-NC of NOZ’ in Fig. 6C in our other article in Oncotarget (https://doi.org/10.18632/oncotarget.18204) are indeed the same and this image belongs to the ‘si-NC of NOZ/Dox’. During that time, we were working on several projects simultaneously and the error should be occurred in the data storage. And we had contacted the editorial office of Oncotarget to explain and correct this error.

Besides, in order to make sure no further problems will be pointed out after the correction of this time, we have self-examined all the raw data of all the projects conducted simultaneously during that time for article [1]. And we found three images of p62 in ‘GBC-SD/Dox Lv-control + Dox’ and ‘GBC-SD/Dox Lv-shRNA + Dox’, ATG5 in ‘GBC-SD/Dox Lv-control + Dox’ in Fig. 8C are not from the corresponding group. For the cause of this, we think it should be occurred in the data storage. And we would like to correct the three images this time together. The correct raw images of these three groups are attached.

**Fig. 8:**
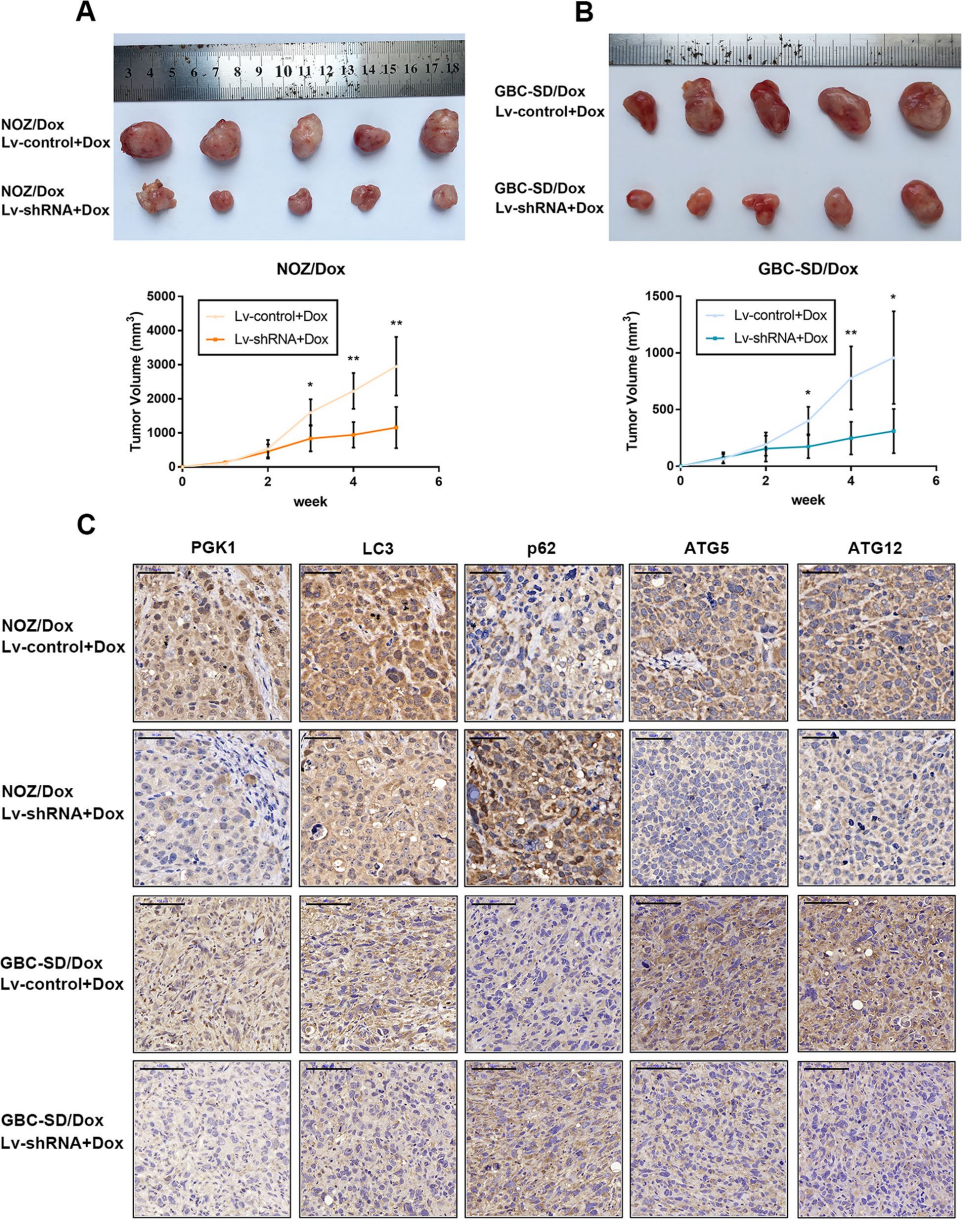
Knockdown of GBCDRlnc1 inhibits autophagy and improves the sensitivity of gallbladder cancer cells to Dox in vivo. **a** The nude mice carrying tumors from NOZ/Dox under different transfection with Dox were shown. Tumor growth curves were calculated per week. **b** The nude mice carrying tumors from GBC-SD/Dox under different transfection with Dox were shown. Tumor growth curves were calculated per week. **c** The PGK1, LC3, p62, ATG5 and ATG12 expression and positive cell numbers was determined by immunohistochemical staining. Scale bar = 50 μm (NOZ/Dox) or 100 μm (GBC-SD/Dox). The mean ± SD of triplicate experiments were plotted, *P < 0.05, **P < 0.01, ***P < 0.001
