# Supplementary figures and images for: Long non-coding RNA GBCDRlnc1 induces chemoresistance of gallbladder cancer cells by activating autophagy
Source: Mol Cancer. 2019 Apr 5;18:82. doi: 10.1186/s12943-019-1016-0 (PMC6449938; doi:10.1186/s12943-019-1016-0)

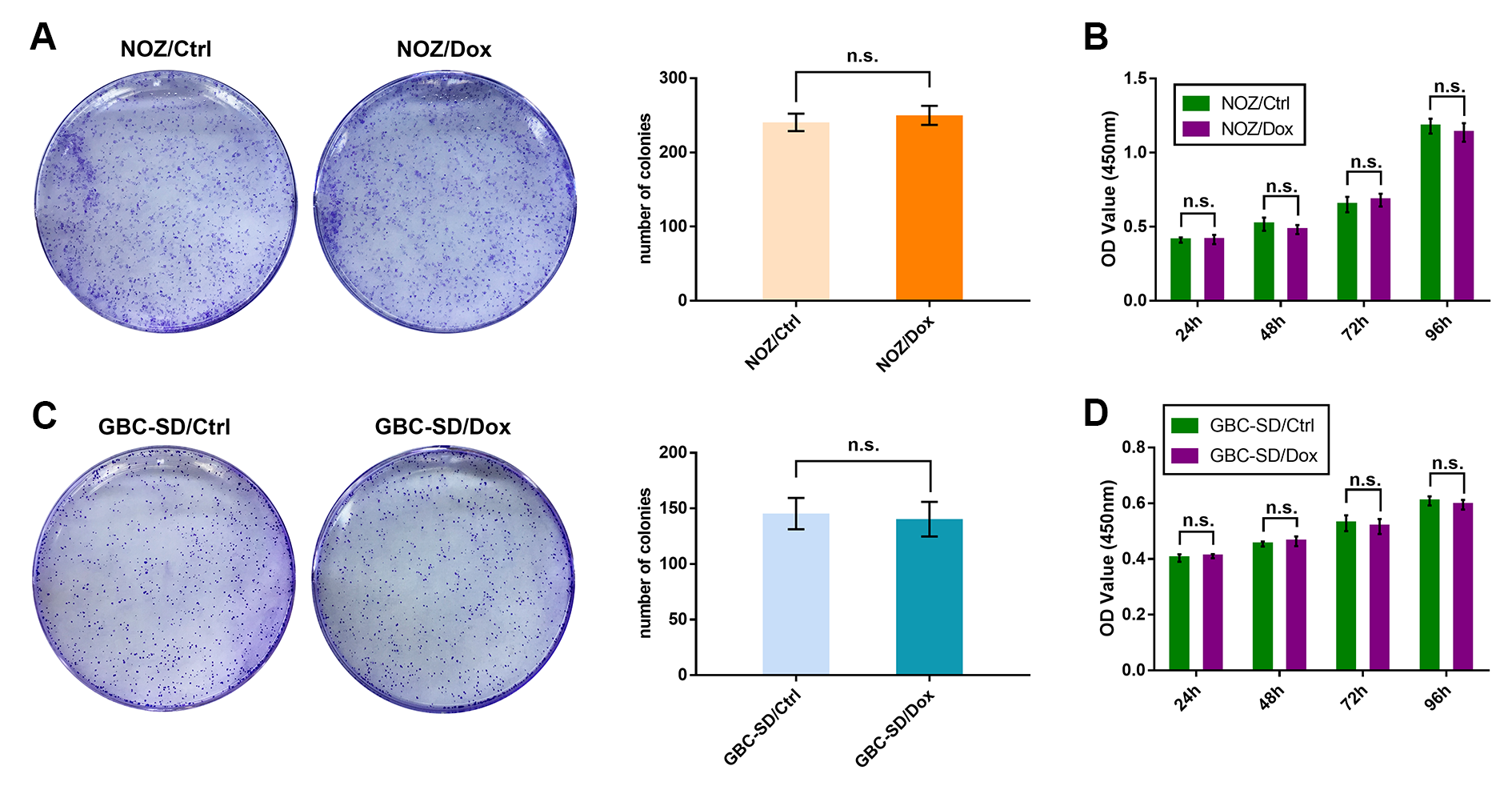

Supplement: Supplementary file 2 — Figure S1. There is no difference in cell proliferation between Dox-resistant gallbladder cancer cells and their parental cells. (A) The coloning ability of NOZ/Dox and NOZ/Ctrl cells was determined by colony formation assay. (B) The cell viability of NOZ/Dox and NOZ/Ctrl cells was determined by CCK8 assay. (C) The coloning ability of GBC-SD/Dox and GBC-SD/Ctrl cells was determined by colony formation assay. (D) The cell viability of GBC-SD /Dox and GBC-SD /Ctrl cells was determined by CCK8 assay. The mean ± SD of triplicate experiments were plotted, n.s., not statistically significant. (TIF 5491 kb) [file 12943_2019_1016_MOESM2_ESM.tif]

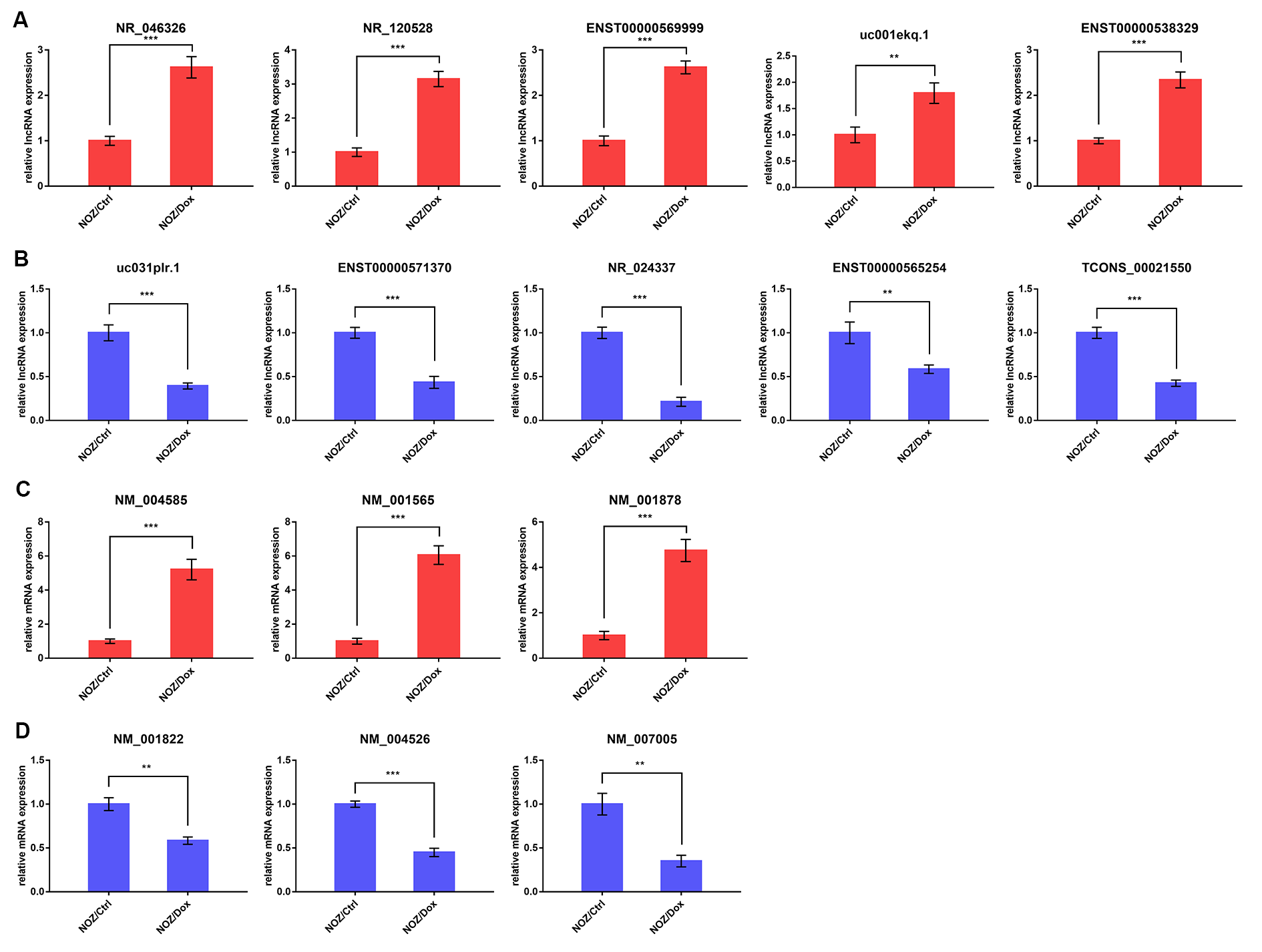

Supplement: Supplementary file 5 — Figure S2. Expression levels of 10 lncRNAs (A-B) and 6 mRNAs (C-D) by qRT-PCR in NOZ/Dox and NOZ/Ctrl cells. The mean ± SD of triplicate experiments were plotted, **P < 0.01, ***P < 0.001. (TIF 7922 kb) [file 12943_2019_1016_MOESM5_ESM.tif]

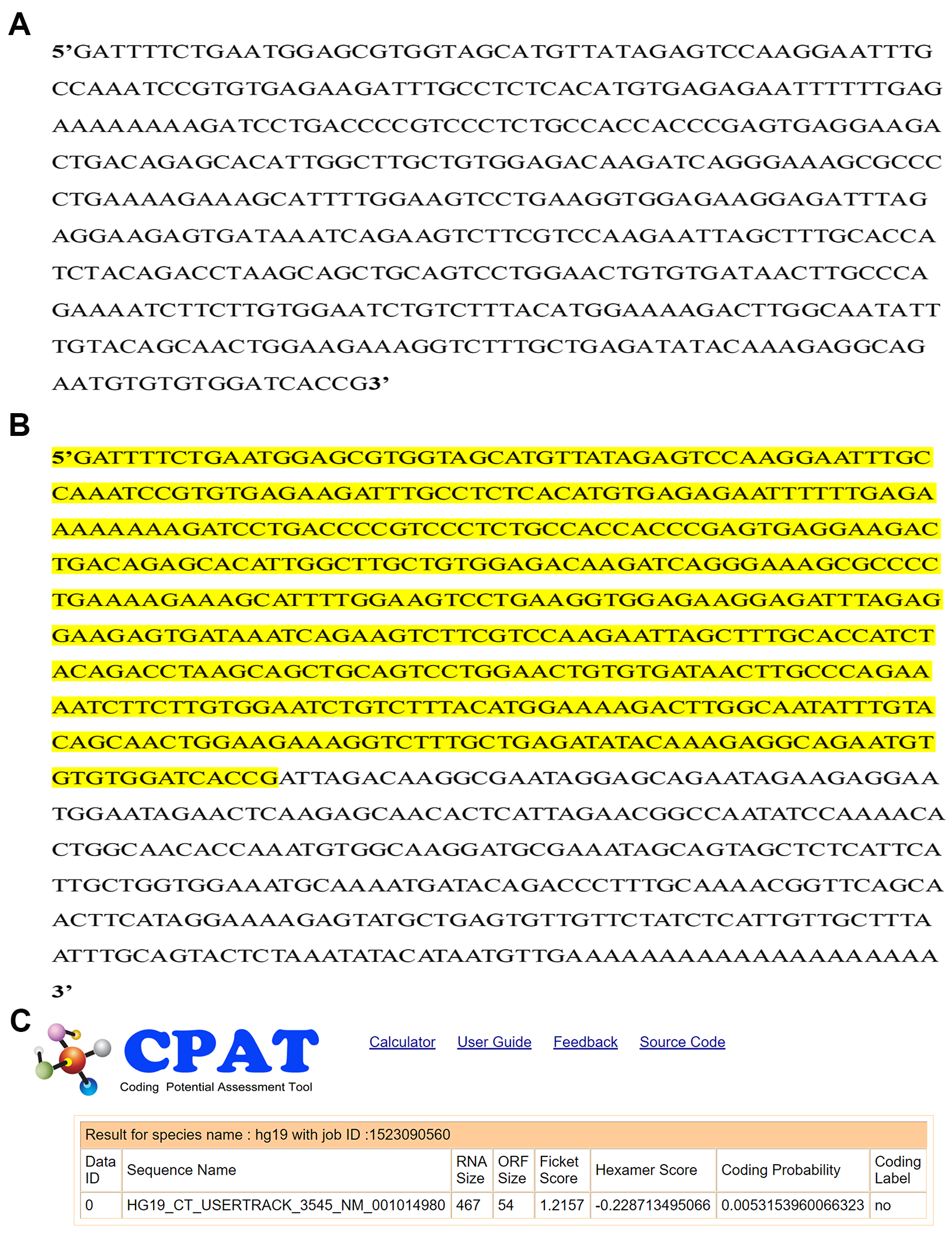

Supplement: Supplementary file 6 — Figure S3. The information of GBCDRlnc1. (A) Nucleotide sequence of the full-length human GBCDRlnc1 gene. (B) The full-length GBCDRlnc1 sequence (highlighted) cloned from 3′-RACE. (C) The coding potential assessment tool showed that GBCDRlnc1 lacks protein-coding potential (the coding probability more than 0.364 is deemed to be able to code protein). (TIF 10162 kb) [file 12943_2019_1016_MOESM6_ESM.tif]

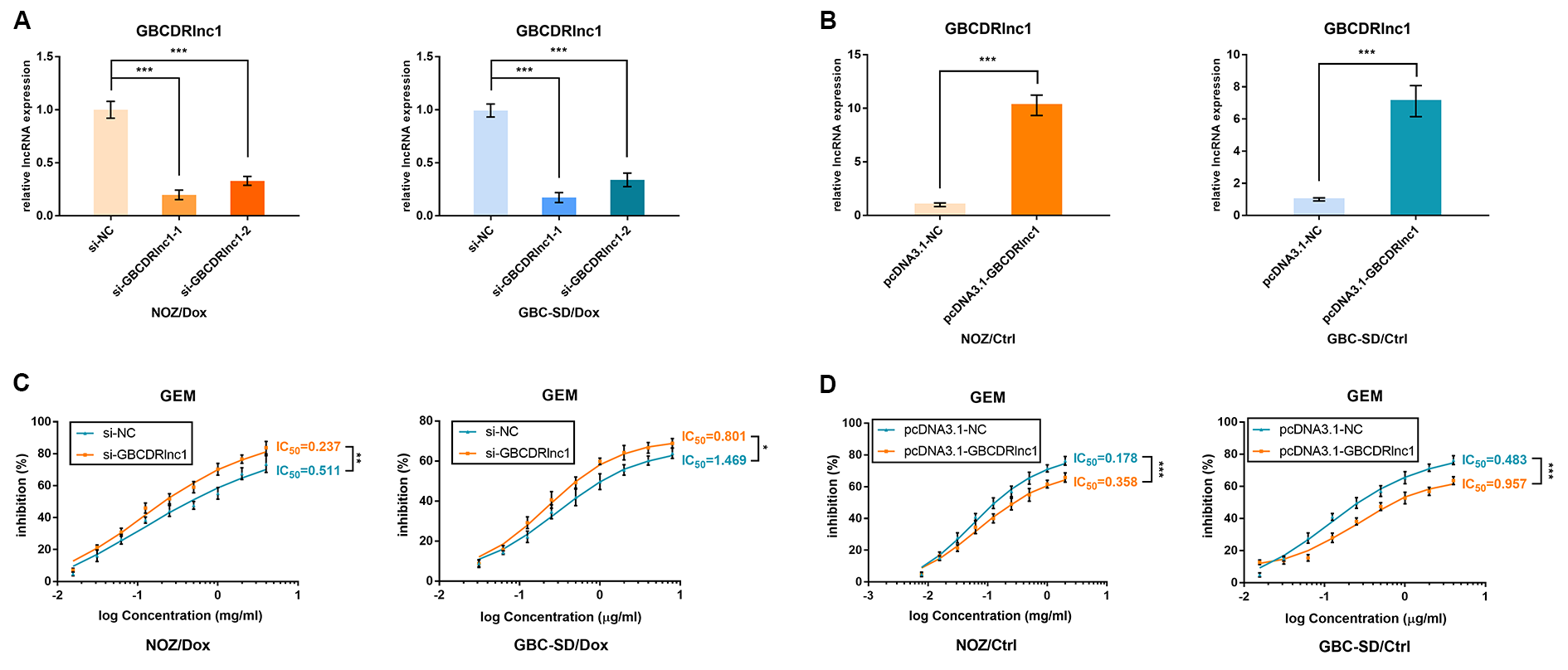

Supplement: Supplementary file 7 — Figure S4. The efficiency of GBCDRlnc1 expression regulation of gallbladder cancer cells in vitro. (A) Relative expression of GBCDRlnc1 in Dox-resistant gallbladder cancer cells with si-GBCDRlnc1 was determined by qRT-PCR. (B) Relative expression of GBCDRlnc1 in Dox-resistant gallbladder cancer cells with pcDNA3.1-GBCDRlnc1 was determined by qRT-PCR. (C) The sensitivities of Dox-resistant gallbladder cancer cells under different transfection with GEM were determined by CCK-8 assay. (D) The sensitivities of the parental gallbladder cancer cells under different transfection with GEM were determined by CCK-8 assay. The mean ± SD of triplicate experiments were plotted, *P < 0.05, **P < 0.01, ***P < 0.001. (TIF 4499 kb) [file 12943_2019_1016_MOESM7_ESM.tif]

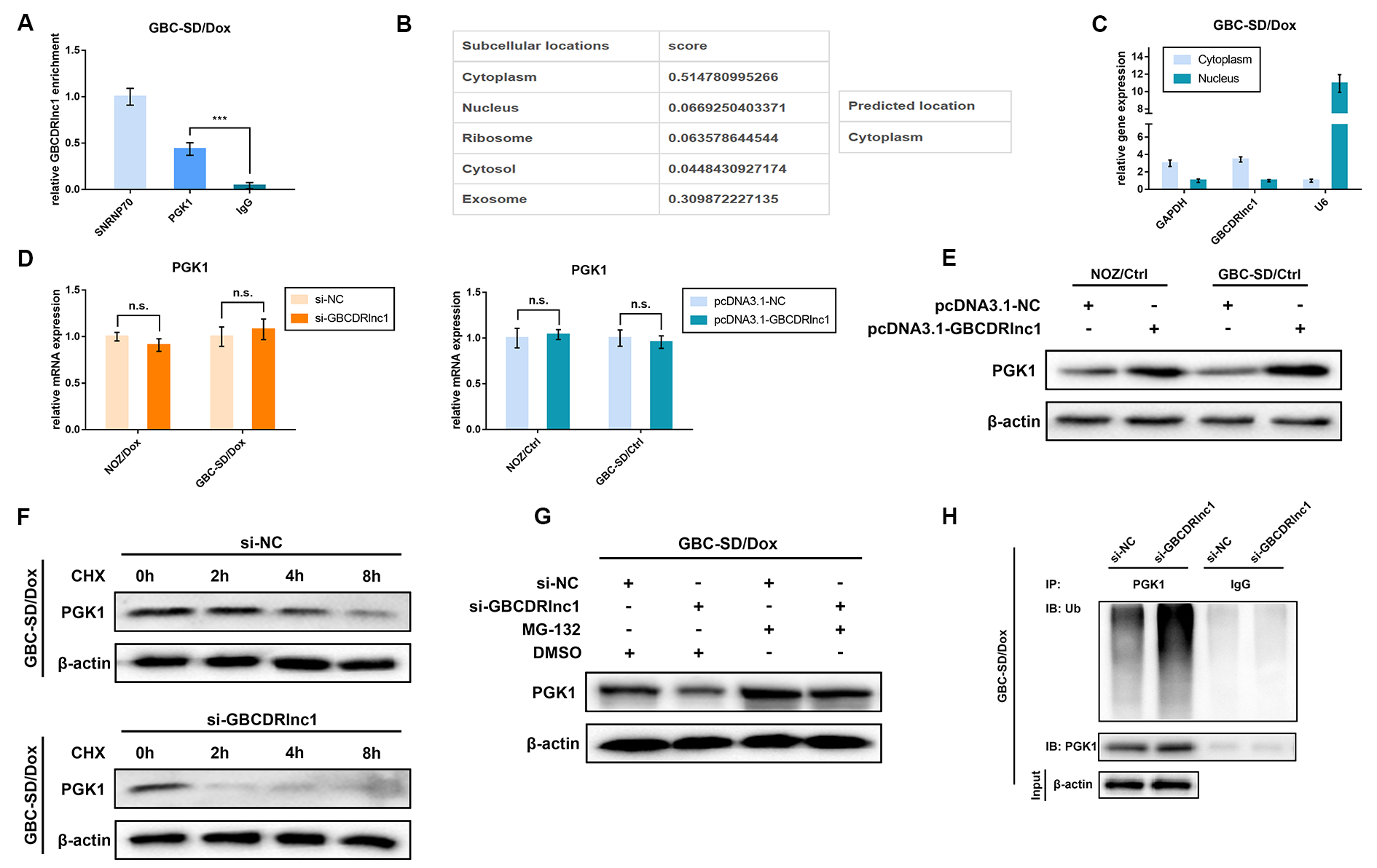

Supplement: Supplementary file 9 — Figure S5. GBCDRlnc1 inhibits PGK1 ubiquitination in gallbladder cancer cells in vitro. (A) Amount of GBCDRlnc1 bound to SNRNP70 (a positive control), PGK1 or IgG (a negative control) was determined by qRT-PCR after RIP in GBC-SD/Dox cells. (B) The online software lncLocator was used to predict the location of GBCDRlnc1. (C) Relative expression of GBCDRlnc1 in cell cytoplasm or nucleus of GBC-SD/Dox cells was determined by qRT-PCR. (D) Relative expression of PGK1 in Dox-resistant gallbladder cancer cells under different transfection was determined by qRT-PCR. (E) The protein levels of PGK1 in the parental gallbladder cancer cells under different transfection were determined by western blot assay. (F) The protein levels of PGK1 in GBC-SD/Dox cells under different transfection with CHX (20 mg/ml) were determined by western blot assay. (G) The protein levels of PGK1 in GBC-SD/Dox cells under different transfection with MG-132 (5 μM) were determined by western blot assay. (H) GBC-SD/Dox cells under different transfection were treated with MG-132 (5 μM) for 24 h. Cell lysates were immunoprecipitated with antibodies against PGK1 or IgG. The levels of ubiquitination were analysed by western blot. Bottom, input from cell lysates. The mean ± SD of triplicate experiments were plotted, ***P < 0.001, n.s., not statistically significant. (TIF 6627 kb) [file 12943_2019_1016_MOESM9_ESM.tif]

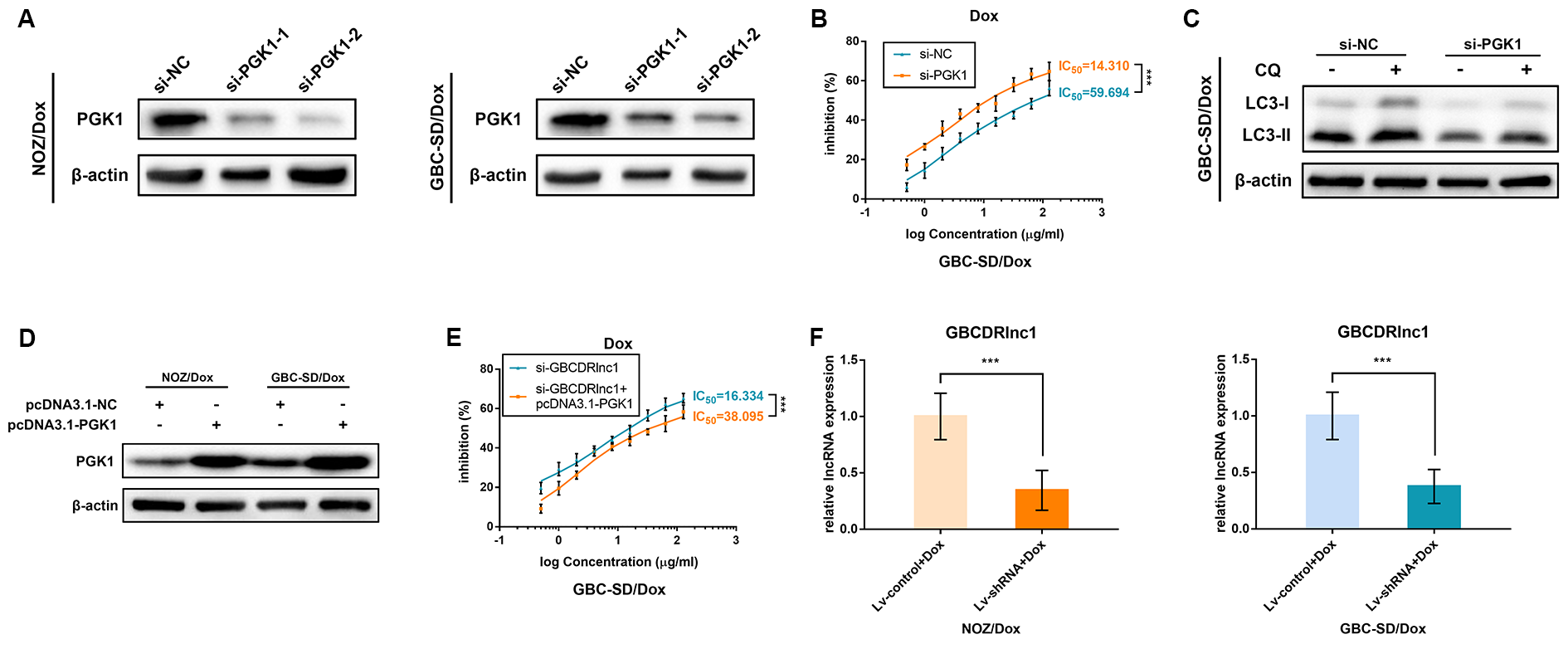

Supplement: Supplementary file 10 — Figure S6. Knockdown of PGK1 suppresses autophagy-associated chemoresistance of gallbladder cancer cells in vitro. (A) The protein levels of PGK1 in Dox-resistant gallbladder cancer cells under different transfection were determined by western blot assay. (B) The sensitivities of GBC-SD/Dox cells under different transfection with Dox were determined by CCK-8 assay. (C) The protein levels of LC3 in GBC-SD/Dox cells under different transfection with CQ (10 μM) were determined by western blot assay. (D) The protein levels of PGK1 in Dox-resistant gallbladder cancer cells under different transfection were determined by western blot assay. (E) The sensitivities of GBC-SD/Dox cells under different transfection with Dox were determined by CCK-8 assay. (F) Relative expression of GBCDRlnc1 in mouse tumor tissues under different transfection with Dox was determined by qRT-PCR. The mean ± SD of triplicate experiments were plotted, ***P < 0.001. (TIF 4358 kb) [file 12943_2019_1016_MOESM10_ESM.tif]
